# Regulation of Key Immune-Related Genes in the Heart Following Burn Injury

**DOI:** 10.3390/jpm12061007

**Published:** 2022-06-20

**Authors:** Jake J. Wen, Keyan Mobli, Geetha L. Radhakrishnan, Ravi S. Radhakrishnan

**Affiliations:** 1 Department of Surgery University of Texas Medical Branch, Galveston, TX 77550, USA; kemobli@utmb.edu; 2 Department of Pediatrics, University of Texas Medical Branch, Galveston, TX 77550, USA; glradhak@utmb.edu

**Keywords:** burn injury, microarray, inflammatory cascade, cardiac dysfunction, cytokines

## Abstract

Immune cascade is one of major factors leading to cardiac dysfunction after burn injury. TLRs are a class of pattern-recognition receptors (PRRs) that initiate the innate immune response by sensing conserved molecular patterns for early immune recognition of a pathogen. The Rat Toll-Like Receptor (TLR) Signaling Pathway RT² Profiler PCR Array profiles the expression of 84 genes central to TLR-mediated signal transduction and innate immunity, and is a validated tool for identifying differentially expressed genes (DEGs). We employed the PCR array to identify burn-induced cardiac TLR-signaling-related DEGs. A total of 38 up-regulated DEGs and 19 down-regulated DEGs were identified. Network analysis determined that all DEGS had 10 clusters, while up-regulated DEGs had 6 clusters and down-regulated DEGs had 5 clusters. Kyoto Encyclopedia of Genes and Genomes (KEGG) analysis showed that DEGs were involved in TLR signaling, the RIG-I-Like receptor signaling pathway, the IL-17 signaling pathway, and the NFkB signaling pathway. Function analysis indicated that DEGs were associated with Toll-like receptor 2 binding, Lipopeptide binding, Toll-like receptor binding, and NAD(P)+ nucleosidase activity. The validation of 18 up-regulated DEGs (≥10-fold change) and 6 down-regulated DEGs (≤5-fold change) demonstrated that the PCR array is a trusted method for identifying DEGs. The analysis of validated DEG-derived protein–protein interaction networks will guide our future investigations. In summary, this study not only identified the TLR-signaling-pathway-related DEGs after burn injury, but also confirmed that the burn-induced cardiac cytokine cascade plays an important role in burn-induced heart dysfunction. The results will provide the novel therapeutic targets to protect the heart after burn injury.

## 1. Introduction

Myocardial dysfunction is a major determinant of multiple-organ failure and mortality in burn injury patients [1,2]. A common cellular response of the injuries is the secretion of inflammatory cytokines from cardiomyocytes [3,4]. Further, the secretion of TNF-α, IL-1β, and IL-6 after burn injury in cardiomyocytes has been shown to produce compartmental and myocardial tissue levels of cytokines that are higher than the cytokine levels measured in the systemic circulation [3]. The specific mechanisms by which inflammatory cytokines alter myocardial function remain unclear, but they are thought to play a role in the hyperdynamic state and cardiomyocyte remodeling [5,6]. Recent attention has been focused on the study of potential mechanisms after burn injury in our lab [7,8,9,10,11]. This study will concentrate on the Toll-like receptor (TLR)-signaling-related differentially expressed genes (DEGs) after burn injury to elucidate the cardiac inflammatory responses to burn injury and disease.

TLRs are one of the most important class of pattern-recognition receptors (PRRs) and are crucial in the regulation of the innate immune system [12]. They function as key receptors in the recognition of pathogen-associated molecular patterns (PAMPs) and damage-associated pattern molecules (DAMPs). This leads to the activation of the innate immune cells in the immune cascade [13]. It is accepted that there is increased TLR expression in burn injury patients [14,15]. These signaling pathways also contribute to inflammation, causing tissue damage. Altering these targets can limit inflammation and fibrosis, but risks infection and inhibited healing [16]. They are a possible molecular target to treat hypertrophic scarring. TLR signaling activates the expression of cytokines and adhesion molecules. These frequently promote leukocyte recruitment and activation [17]. To date, there have been 13 TLRs identified in mice/rats and 10 in humans. Ligand binding to TLRs activates the transcription factors CREB, AP-1, NFκB, IRF3, and IRF7 [18]. However, a comprehensive understanding of TLR-signaling cascades in burn injury is not yet established. In this study, RNA qRT-PCR array technology was utilized to evaluate the relevant expression of TLR-regulated genes in cardiac immune cells from burn injury rats. We aimed to identify the key TLR pathways that may serve as therapeutic targets for treating burn-injury-induced heart dysfunction.

In many clinical studies, the size of burn injury correlates with the level of circulating DAMPs and cytokines. In addition, in early 2-week post-burn-injury, serum levels of decorin, a TLR2 and TLR4 ligand, were a factor (along with early interleukin [IL]-1β and late transforming growth factor [TGF]-β) that was suggested to predict hypertrophic scar formation. Thus, reducing DAMP- and PAMP-TLR signaling can potentially improve burn outcomes by mitigating injury progression, systemic inflammation, and the prolonged inflammation and healing associated with hypertrophic scarring.

The rat TLR Signaling Pathway RT² Profiler PCR Array profiles the expression of 84 genes central to TLR-mediated signal transduction and innate immunity. This array includes members of the TLR-signaling family as well as adaptor and effector proteins. Members of the NFκB, JNK/p38, IRF and JAK/STAT signaling pathways downstream of TLR activation are also included in our array. Using real-time PCR, we can easily and reliably analyze the expression of a focused panel of genes related to TLR-mediated signal transduction with this array.

This study tested the hypothesis that the increases in immune cells in burn-induced cardiac tissues play an important role the development of burn-injury-sensitive heart damage in male Sprague Dawley rats. Our initial experiments compared the influence of burn injury on cardiac mitochondrial responses in the Sprague Dawley (SD) rat, which demonstrates mitochondrial-dependent cardiac damage [7,9,10,11]. Burn injury involves an inflammatory mechanism, as previously described by Laszlo M. Hoesel et al. [19]. A further analysis of these cardiac cells then used a PCR profiler array of TLR signaling to assess one of the primary activation pathways of these cells (Figure S1). Transcriptional profiling specific to 84 TLR pathway-associated genes (Figure S1) was performed and compared to the corresponding sham control. The aim of this analysis was to identify burn-injury-associated TLR pathway genes by comparison of burn-injury and non-burn-injury expression patterns, particularly regarding the identification of biomarkers for burn-induced cardiac damage. Finally, functional experiments were performed to validate the identified TLR-related genes.

## 2. Materials and Methods

### 2.1. Ethics Statement

The study was approved by the Ethics Committee of the University of Texas Medical Branch (Galveston, TX, USA) and by our Institutional Animal Care and Use Committee (IACUC, Protocol number: 1509059).

### 2.2. Animals, Burn Preformation and Sample Collection

Male Sprague Dawley rats weighing 250–300 g were purchased from Harlan Laboratories (Indianapolis, IN, USA). Rats were kept in our animal housing to adapt for one week before the procedure. They were maintained on a light–dark cycle (12 h:12 h; ~25 °C), and freely took food and water during the study. A well-established model in our lab for the induction of a 60% total body surface area (TBSA) full-thickness burn was utilized [7,8,9,10,11]. Briefly, rats were given analgesia (buprenorphine, 0.05 mg/kg) by subcutaneous injection and anesthetized with isoflurane (3–5%), then submerged in near-boiling water (95–98 °C) to cause burn injury of 30% TBSA of the dorsum (10 s) and 30% TBSA of the abdomen (2 s). Subsequently, the burned rats (burned group) were immediately resuscitated with lactated Ringer (LR) solution (40 mL/kg, i.p.) and oxygen. Humanely, analgesia (buprenorphine, 0.05 mg/kg) was given at 6 h post-burn (hpb) by subcutaneous injection. At 24 hpb, rats were humanely euthanized, and tissue was collected into one microtube, flash frozen in liquid nitrogen and stored at −80 °C for later processing.

### 2.3. RNA Isolation

Total RNA from each rat heart tissue (10 mg) was isolated using RNeasy Mini Kit (Qiagen, Gaithersburg, MD, USA, Cat# 74104) according to the manufacturer’s instructions. Slight modifications in DNase I, RNase-free (Westlake, LA, USA, Cat# M0303S) treatment were used to digest contaminated genomic DNA. For judging the integrity and overall quality of isolated RNAs, 2 µg RNAs were run on 1% native agarose gels. A spectrophotometer (The DU^®^ 700 UV/Visible, Beckman Coulter, Pasadena, CA, USA), NanoDrop (NanoDrop ND-1000 Spectrophotometer), and Agilent 2100 Bioanalyzer were applied to measure RNA quality by determination of absorbance at 260 and 280 nm (OD260/280 ratio ≥ 2, 1 OD260 Unit = 40 µg/mL RNA), and the ratio of 28 S to 18 S rRNA (≥1.7), separately.

### 2.4. First-Strand cDNA Synthesis

Two µg RNA was utilized to make cDNA using a SuperScript^®^ III Reverse Transcriptase (Invitrogen), Waltham, MA, USA, following the company manual. The cDNA synthesis mixture (13 μL) containing 2 μg of total RNA, 1 μL of 500 ng of oligo(dT)18, 1 μL of 10 mM dNTP mix, and sterile RNase-free water was heated to 65 °C for 5 min to denature the RNA, and incubated on ice for 1 min. A total of 4 μL of 5× first-strand buffer, 1 μL of 0.1 M DTT, 1 μL of RNase inhibitor, and 1 μL of SuperScript III reverse transcriptase were added to the reaction tube after centrifugation. This was then mixed by pipetting gently and incubated at 42 °C for 60 min. The reaction was inactivated by heating at 95 °C for 15 min, and diluted to a final volume of 102 μL with ddH_2_O to be stored at −80 °C. The cDNA was used as a template to run Real-Time PCR array and qPCR using iCycler Thermal Cycler (Bio-Rad, Hercules, CA, USA) with SYBR-Green Supermix (SYBR Green Real-Time PCR Master Mixes, ThermoFisher, Sugar Land, TX, USA, Cat# 4309155).

### 2.5. Real-Time RT² Profiler PCR Array (QIAGEN, Cat. no. PARN-018Z)

The high quality cDNAs were used on the real-time RT² Profiler PCR Array in combination with RT² SYBR^®^ Green qPCR Mastermix (Cat. no. 330529). The PCR Array was used to identify and pool the differentially expressed gene profiles that were related to TLR pathways and profile gene expression, according to the instructions from the manufacturer. RNA from two randomly selected rats in each group was converted to cDNA and then amplified by PCR. The means were used for data analysis. The PCR array was performed in triplicate. The delta threshold cycle (Ct) was calculated by subtracting Ct for the average of 6 housekeep genes, including β-actin (H1), β-2-macroglobulin (H2), GAPDH (H3), hypoxanthine phosphoribosyl transferase 1 (H4), ribosomal protein lateral stalk subunit P0 (H5), and (R)-2-hydroxyglutaryl-CoA dehydratase-activating ATPase (H6) from Ct for genes of interest. The rat profile PCR array profiles the expression of 84 key genes associated with the TLR-signaling pathway as well as related pathway-activity signature genes. In brief, a reaction mixture was prepared by successively adding 550 μL of 2× RT^2^ SYBR^®^ Green qPCR Mastermix, 102 μL of the diluted first-strand cDNA synthesis reaction, and 448 μL of ddH_2_O. The mixture was then administrated to the PCR array and PCR performed on a Bio-Rad^®^ iCycler at 95 °C for 10 min, 40 cycles of 95 °C for 15 s, and 60 °C for 1 min.

### 2.6. Analysis of Real-Time RT² Profiler PCR Array

Online Analysis Software (https://geneglobe.qiagen.com/us/analyze (accessed on 6 June 2021)) was used to analyze PCR Array data. All plates had 3 positive PCR controls and 3 reverse-transcription controls. Calculations of contamination with rat genomic DNA, according to the manufacturer’s instructions, showed the presence of genomic DNA in an acceptable range that would not influence experiment performance. Values of the cycle threshold (Ct) obtained in quantification were used for calculations of folds changes in mRNA abundance, according to the 2^−ΔΔCt^ method [20]. The average was chosen from the group of six housekeeping genes as the varying reference gene [21]. Changes in mRNA level for evaluated genes were assessed in all groups in relation to the control group of animals, with mRNA abundance set up arbitrarily as 1.

### 2.7. String-DB Analysis of DEGs

After opening the String-DB website (https://string-db.org/ (accessed on 6 June 2021)) and inserting all derived protein names in Multiple Proteins by Names/Identifiers under SEARCH, we automatically obtained the data by following the website’s instructions. The results from String-DB included more than 10 parameters, such as Kyoto Encyclopedia of Genes and Genomes (KEGG) pathway, function, compartments, component, InterPro, clusters, etc. These are summarized in Tables S1–S3.

### 2.8. Signaling Pathway Mapping for DEGs

The Kyoto Encyclopedia of Genes and Genomes (KEGG) Mapper (https://www.genome.jp/dbget-bin/www_bget?rno (accessed on 3 March 2021)) was used to analyze differentially expressed genes. The ratio mean of gene expression was calculated in the control and burn groups, which was visualized in a color code (reds for up-regulated DEGs and blues for down-regulated DEGs) in the top four pathways, including TLR-signaling pathway (KEGG rno04620), IL-17 signaling pathway (KEGG rno04657), RIG-I-like receptor signaling pathway (KEGG rno04622), and NfkB signaling pathway (KEGG rno04064).

### 2.9. Validation of Identified Genes by qPCR

Total RNA isolation and cDNA production were similar to the methods described in 2.3 and 2.4. A total of 25 µL of reaction mixture was prepared by successively adding 12.5 μL of 2× RT2 SYBR^®^ Green qPCR Mastermix, 2 μL of the diluted first-strand cDNA synthesis reaction, 0.6 µL of primer mixture and 9.9 μL of ddH_2_O. The mixture was then administrated to the PCR tube and PCR was performed on a Bio-Rad^®^ iCycler at 95 °C for 10 min, 40 cycles of 95 °C for 15 s, and 60 °C for 1 min. The results were analyzed using the ΔΔCt method. The primers we designed were based on identified DEGs using NCBI’s Primer-BLAST. The primers for qPCR tests used in the study are shown in Table 1. The threshold cycle (Ct) values of target genes were normalized to the housekeeping GAPDH gene and calculated as fold change.

### 2.10. Protein–Protein Interaction (PPI) Networks

Protein–protein interaction networks of differentially expressed proteins (DEPs) were constructed using STRING 9.1 (http://string-db.org/ (accessed on 6 June 2021)). The name of each individual protein was given as a query to the STRING database, and the corresponding PPI information was retrieved by enabling different prediction methods. The networks were made with a confidence cutoff of ≥0.45.

### 2.11. Statistical Analysis

Data are presented as the mean ± standard deviation or a number (%). Statistical analysis was performed with the use of GraphPad 9.3 software package (GraphPad Holdings, LLC, San Diego, CA, USA). A two-tailed Student’s *t*-test was applied for this analysis. Before the Student’s t-test, the Fisher–Snedecor test was used to verify the hypothesis about equality of variances. Unequal variances were not found. Therefore, a t-test with Welch correction was not used in the project. Significance is expressed with * (24 hpb rats vs. sham rats) (* *p* < 0.05, ** *p* < 0.01, *** *p* < 0.001). Regarding PCR array data, the data analysis was conducted using the ΔΔCt module on the Qiagen GeneGlobe Data Analysis Center portal. The efficiency of all the primers used in the kits was shown to be over 90%. The RT-PCR arrays contained control wells/samples for the determination and/or verification of rat genomic contamination, reverse transcription control, and positive PCR controls. At least six reference genes were used for data normalization. In these analyses, genes with a greater than 1.5-fold change in expression at a value of *p* < 0.05 between burn- and sham-group rats were defined as differentially expressed and selected for inclusion in comparative analyses. Gene Ontology (GO) analysis was conducted using String-DB resources (http://string-db.org (accessed on 6 June 2021)).

## 3. Results

### 3.1. Highly Qualified RNA

**Figure 1:**
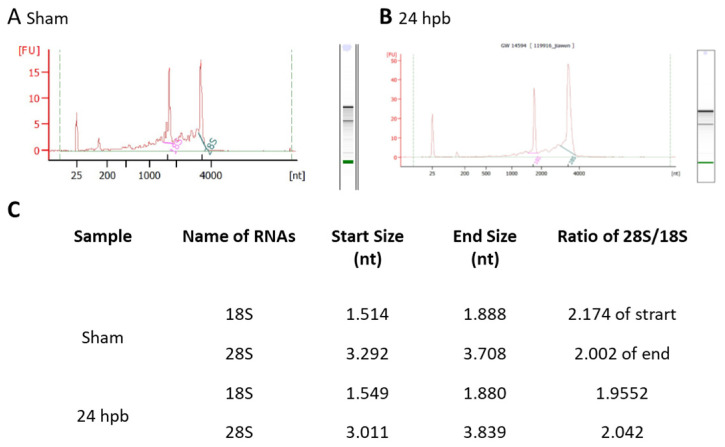
Highly intact RNA: RNA was purified from heart tissues using the Qiagen RNeasy Mini Kit. The purified RNA was analyzed using a Beckman 700 system (ratio of 28 S to 18 S rRNA: ≥1.55), NanoDrop 2000, and Agilent 2100 Bioanalyzer (ratio of 28 S to 18 S rRNA: ≥1.7). A high RNA integrity number (RIN) of 9.6 was obtained, indicating highly intact RNA. (**A**) sham; (**B**) 24 hpb; (**C**) real values from Agilent 2100 Bioanalyzer.

Highly qualified/purified RNA is the bottleneck for the transcriptome/microarray/RT-PCR array profile. We used different methods to test our RNA quality, including agarose electrophoresis (data not shown), a spectrophotometer, NanoDrop, and an Agilent 2100 Bioanalyzer. Figure 1 shows the qualities and purities of isolated sham RNA (Figure 1A) and 24 hpb RNA (Figure 1B). They were satisfactory to move forward, as evidenced by the ratios of 28 S:18 S of ≈2 (Figure 1C).

### 3.2. PCR Array in Cardiomyocytes

**Figure 2:**
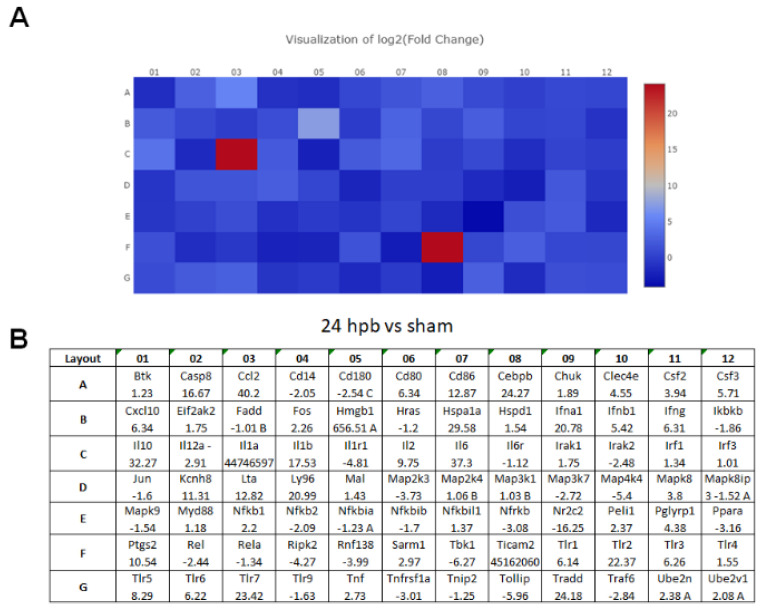
(**A**) Heat maps of real-time RT 2 Profiler PCR Array showing differential expression of 84 TLR-related genes stimulated by burn injury. (**B**) The ≥1.5-fold changes represent the genes that are up-regulated, and the ≤−1.5-fold changes represent the genes that are down-regulated by burn injury.

**Figure 3:**
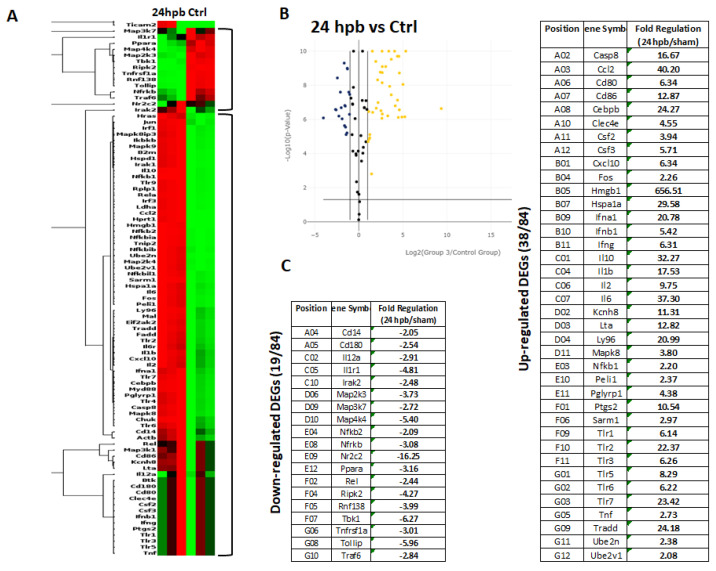
Expression of TLR-Related Genes in burn injury rats. Sham control or burn injury analyzed through a Rat TLR RT2 Profiler PCR Array (QIAGEN): (**A**) Heatmap displaying hierarchical clustering of the entire dataset of expressed genes and indicating co-regulated genes across sham control and burn experimental groups: healthy sham control rats—CTRL; burn injury rats—24 hpb. Clustering analysis showed that genes upregulated in burn injury rats belong to apoptosis modulation, IL-1 signaling, TLR signaling, RIG-I-like receptor signaling and IL-17 signaling pathways; (**B**) The volcano plots identify significant changes in gene expression between groups. The volcano plot displays the statistical significance (*p* < 0.05) versus fold change on the y and x axes, respectively; (**C**) Venn diagram and list of TLR-related genes regulated in burn injury rats. The expression levels of genes are presented as fold-regulation values (those greater than 1.5 are indicated, and those less than 1.5 are indicated for burn injury rats compared to those for controls (24 hpb vs. CTRL column). Complete data are provided in Table S1a–e.

The TLR RT2 Profiler PCR Array had 84 TLR-signaling-pathway-related genes (Figure S1, wells A1 to G12) and 6 housekeeping genes (Figure S1 H1–6), 3 positive genes (Figure S1 H7–9) and 3 negative genes (Figure S1 H10–12). The Qiagen analysis center provided the heatmap of the real-time RT2 Profiler PCR Array (Figure 2A) and the fold changes in burn injury compared to the sham control in each of the 96 wells (Figure 2B). The expression profile of the 84 genes relevant to TLR were graphed on a histogram (Figure S2). The fold-change calculations were conducted using the online software of the Qiagen Analysis Center, comparing the burn injury groups to the sham control groups. Further analysis displayed hierarchical clustering of the expressed genes (Figure 3A), and the volcano plots (Figure 3B) indicate co-regulated genes across the sham control and burn experimental groups. Cardiomyocytes in 24 hpb rats represented 38 predominantly upregulated TLR-pathway-related genes such as CASP8, CCL2, CEBPB, CXCL10, HMGB1, HSPA1A, IFNA1, IFNB1, IL10, IL1A, IL6, LY96, TLR1, 2, 3, 5, 6, 7, TRADD, etc. (38 genes, fold change ≥ 2.0, Figure 3C and Table 2); and 19 down-regulated DEGs such as CD14, CD180, IL1R1, MAP2K3, MAP3K7, MAP4K4, RIPK2, RNF138, TBK1, TOLLIP, etc. (fold change ≤ 2.0, Figure 3C and Table 2). Signaling-pathway analysis showed 14 divisions (Table 3) including TLR signaling (six of eight TLRs were increased in 24 hpb), effectors (only Irak2 ↓), interacting proteins and adaptors (Hspa1a ↑, Cd14 and Tollip ↓), apoptosis (Casp8 ↑), ubiquitin-conjugating pathway (↑ of 12 DEGs and 1 ↓), regulation of adaptive immunity (↑ of six DEGs and ↓ of two DEGs), downstream pathway of Toll-like receptor NFKB signaling (five DEGs ↓, four EDGs ↑), JUK/p38 signaling (three DEGs ↑ and one ↓), JAK/STAT signaling (three DEGs ↑ and one ↓), interferon regulatory factor signaling (four DEGs ↑ and one DEGs ↓), cytokine signaling (two DEGs ↑ and two DEGs ↓), NFkB/IL6 signaling (five DEGs ↑ and one DEG ↓), JUN/MARK signaling (three DEGs ↑ and two DEGs ↓), and others (three DEGs ↑ and five DEGs ↓).

### 3.3. Analysis of Interaction Network and Function Network of Identified Genes after Burn Injury

**Figure 4:**
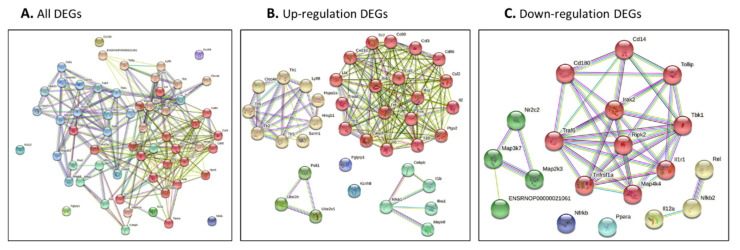
(**A**–**C**) Protein–protein interaction network of all identified genes for burn injury using String-DB. Genes were identified using our network approach. Colored nodes indicate first shell interactions. Edges represent protein–protein associations. Blue indicates that the information is from curated databases, and pink indicates that it is experimentally determined; green is the gene neighborhood; dark blue represents gene co-occurrence; yellow represents information gathered from text mining; black represents co-expression; and light blue represents protein homology. Proteins with no interactions are not included in the figure for clarity. (**A**) all DEGs; (**B**) up-regulated DEGs; and (**C**) down-regulated DEGs. (**A**,**B**) Functional interaction network based on the STRING database and Gene Ontology classification. Different colors represent different clusters, and the details are described in the text.

The genes identified by our network approach were input (Figure 4A for all DEGs, Figure 4B for up-regulated DEGs and Figure 4C for down-regulated DEGs). The colored nodes indicate first shell interactions; the edges represent protein–protein associations; blue indicates that the information is from curated databases and pink indicates that it is experimentally determined; green is gene neighborhood; dark blue represents gene co-occurrence; yellow represents information gathered from text mining; black represents co-expression; and light blue represents protein homology. Proteins with no interactions are not included in the figure for clarity. The functional interaction networks based on the STRING database and kmeans clustering method are shown in different colors. In detail, in Figure 4A, the red nodes correspond to proteins involved in the chemokine-related pathway, forming a multimeric SMAD3/SMAD4/JUN/FOS complex. These play a role in signal transduction; cell proliferation and differentiation; the generation of purine nucleotides through the purine salvage pathway; and regulation of the immune response. The brown nodes correspond to proteins involved in mediating the innate immune response, and recognizing DAMPs and PAMPs; these are multifunctional redox-sensitive proteins with various roles in different cellular compartments, in the protein quality-control system, in ensuring the correct folding of proteins and the re-folding of misfolded proteins, in controlling the targeting of proteins for subsequent degradation, and in the clearance of cytotoxic polyQ protein aggregates. The dark gold rod node corresponds to proteins involved in CD180 binding. The green–yellow nodes correspond to proteins involved in innate immunity and function in the intracellular killing of bacteria. The two green nodes correspond to proteins acting as a growth factor for activated T and NK cells, enhancing the lytic activity of NK/lymphokine-activated killer cells, and stimulating the production of IFN-gamma by resting PBMC. The green nodes correspond to proteins involved in regulating the expression of genes involved in immune and inflammatory responses, playing a significant role in adipogenesis, as well as in the gluconeogenic pathway. The blue nodes correspond to proteins involved in the important repression of nuclear receptor signaling pathways. The light sky-blue nodes correspond to proteins involved in: upstream protease of the activation cascade of caspases, which trigger intracellular signaling cascades, leading to transcriptional up-regulation and mRNA stabilization; various processes such as neuronal proliferation, differentiation, migration and programmed cell death; mediating the ubiquitination of free/unanchored polyubiquitin chains that leads to MAP3K7 activation and activation of NF-kappa-B and JUN; dendritic cell (DC) maturation and/or activation; and the error-free DNA repair pathway, contributing to the survival of cells after DNA damage (Figure 4A and Table S2).

#### 3.3.1. Protein–Protein Interaction Network and Function Network of Identified Up-Regulated Genes after Burn Injury

Figure 4B presents the networks from the burn-injury-upregulated DEGs (fold change > 2.0). By analyzing the functional interaction network, six clusters were obtained (Figure 4B, Table S2). In detail, the red nodes correspond to proteins involved in cell apoptosis/death regulation/activation or that have a role in the proinflammatory cytokine. They may also participate in T-cell effector function, and perhaps in T-cell development, signal transduction, cell proliferation and differentiation, regulation of the immune response, or lymphocyte and monocyte differentiation. The yellow nodes correspond to proteins acting as a pattern-recognition receptor of the innate immune system, recognizing DAMPs and PAMPs, and with various roles in different cellular compartments, replication, transcription, chromatin remodeling, V(D)J recombination, DNA repair, and genome stability. They also play a pivotal role in the protein quality-control system, ensuring the correct folding of proteins and the re-folding of misfolded proteins, and controlling the targeting of proteins for subsequent degradation. The lime green nodes correspond to proteins involved in mediating the transcriptional activation of target genes; these play a role in the control of progress through the cell cycle; in differentiation; and in the error-free DNA repair pathway, with contributions to the survival of cells after DNA damage or protein ubiquitination. The green nodes correspond to proteins involved in potent proinflammatory cytokines, promoting Th17 differentiation of T-cells, and synergizing with IL12/interleukin-12 to induce IFNG synthesis from T-helper 1 (Th1) cells. These include various processes such as neuronal proliferation, differentiation, migration, and programmed cell death, stimulating the stress-activated protein kinase/c-Jun N-terminal kinase (SAP/JNK) signaling pathway. The blue nodes correspond to proteins involved in the potassium voltage-gated channel. The purple nodes correspond to proteins involved in immunity to Gram-positive bacteria by interfering with peptidoglycan biosynthesis (Table S2).

#### 3.3.2. Protein–Protein Interaction Network and Function Network of Identified Down-Regulated Genes after Burn Injury

Similar to Section 3.3.1, down-regulated DEGs were analyzed in the protein–protein interaction network (Figure 4C and Table S3). A functional cluster analysis resulted in five pools. The red nodes correspond to the proteins involved in mediating the innate immune response; the recruitment of adapter molecules, triggering intracellular signaling cascades and leading to transcriptional up-regulation and mRNA stabilization; connecting the ubiquitin pathway to autophagy by functioning as a ubiquitin-ATG8 family adapter and, thus, mediating autophagic clearance of ubiquitin conjugates; the clearance of cytotoxic polyQ protein aggregates; and mediating the ubiquitination of the free/unanchored polyubiquitin chain that leads to MAP3K7 activation and the activation of NF-kappa-B and JUN. The yellow nodes correspond to the proteins that act as a growth factor for activated T and NK cells, enhancing the lytic activity of NK/lymphokine-activated killer cells, and stimulating the production of IFN-gamma with resting PBMC. The green nodes correspond to the proteins that act as a repressor or activator of transcription, an important repressor of nuclear receptor signaling pathways. The cyan nodes correspond to the proteins that are key regulators of lipid metabolism, regulating the peroxisomal beta-oxidation pathway of fatty acids and functioning as transcription activators for the ACOX1 and P450 genes.

### 3.4. The Analysis of Enriched Gene Ontology (GO) for Identified DEGs

Enriched GO is presented as GO molecular functions (GO-MFs), GO cellular components (GO-CCs) and the GO biological processes (GO-BPs). Table 4 reveals the top 10 GO-MFs, top 10 GO-CCs, and top 10 GO-BPs based on the strengths.

### 3.5. The Signaling Pathway Mapping for Identified DEGs

**Figure 5:**
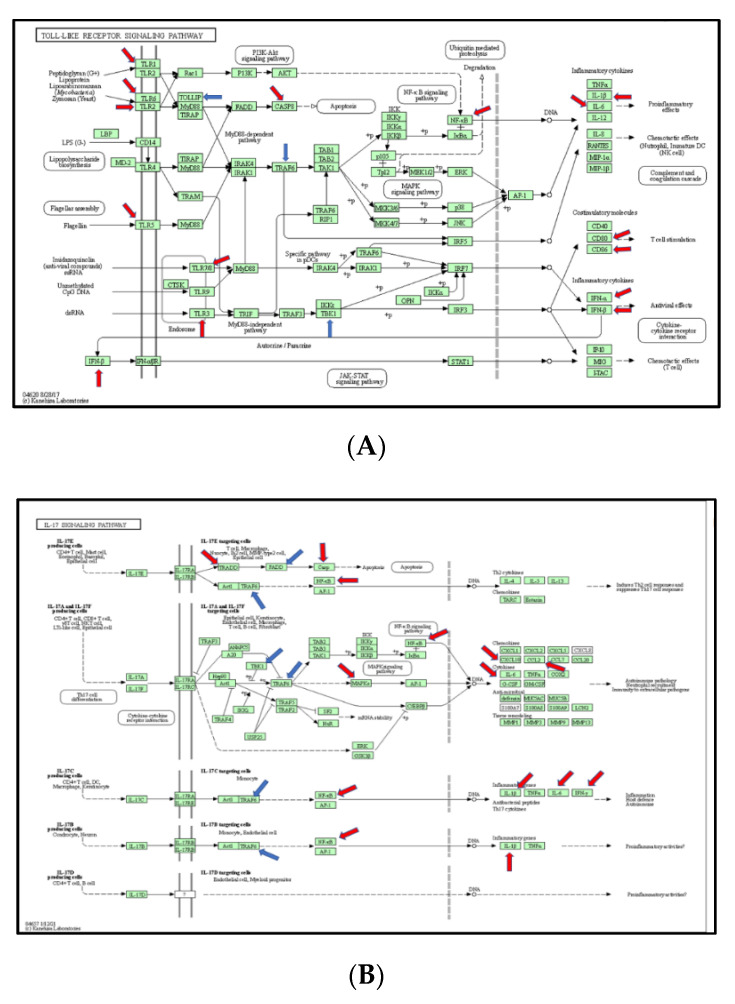

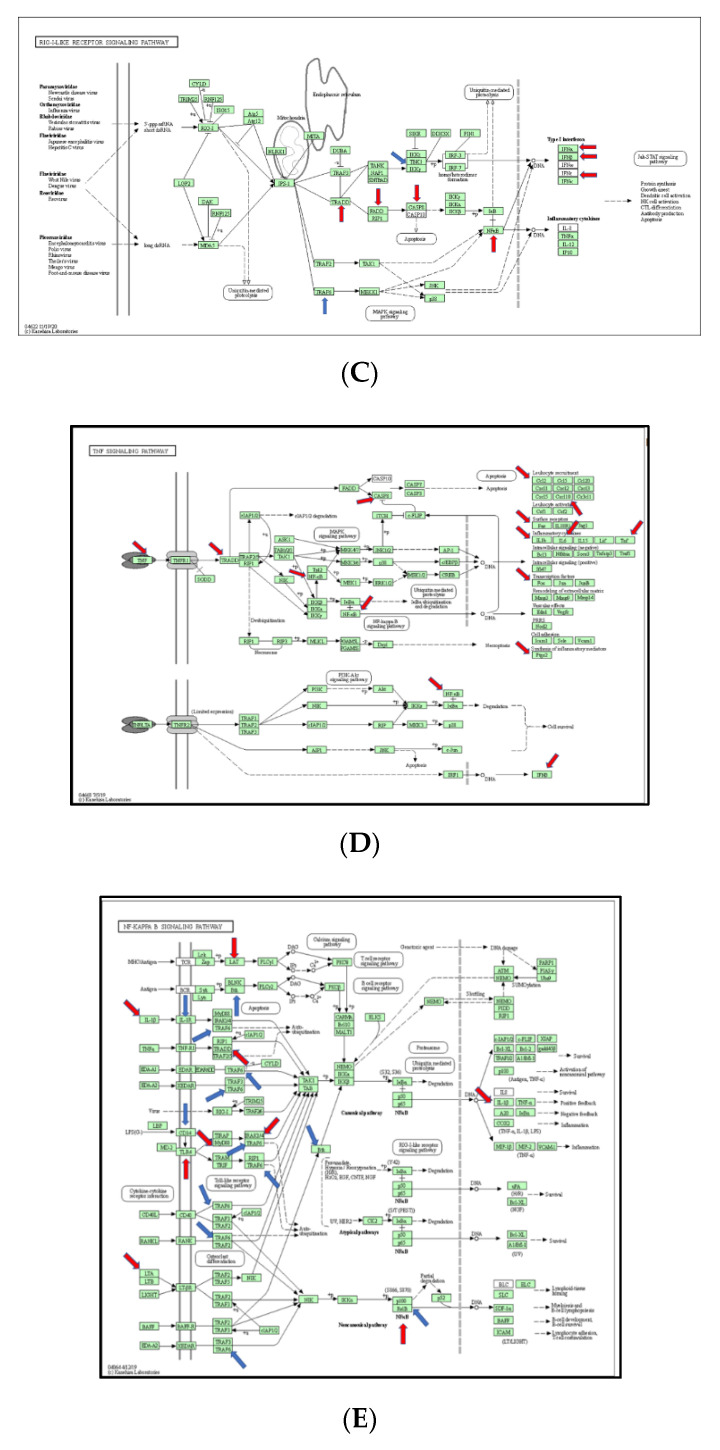
(**A**) Mapping of TLR-signaling pathway in burn injury rats. Each gene in the KEGG TLR-signaling network (KEGG rno04620) is colored according to its expression level in the burn injury group compared with that of the sham group. Genes that were significantly upregulated are colored red, and those that were significantly downregulated are marked blue; (**B**) Mapping of IL-17 signaling pathway in burn injury rats. Each gene in the KEGG TLR-signaling network (KEGG rno04657) is colored according to its expression level in the burn injury group compared with that of the sham group. Genes that were significantly upregulated are colored red, and those that were significantly downregulated are marked blue; (**C**) Mapping of RIG-I-Like receptor signaling pathway in burn injury rats. Each gene in the KEGG TLR-signaling network (KEGG rno04622) is colored according to its expression level in the burn injury group compared with that of the sham group. Genes that were significantly upregulated are colored red, and those that were significantly downregulated are marked blue; (**D**) Mapping of TNF signaling pathway in burn-injury-induced upregulated DEGs in rats. Each gene in the KEGG TNF signaling network (KEGG rno04668) is colored according to its expression level in the burn injury group compared with that of the sham group. Genes that were significantly upregulated are colored red, and those that were significantly downregulated are marked blue; (**E**) Mapping of NfkB signaling pathway in burn injury rats. Each gene in the KEGG NFkB signaling network (KEGG rno04064) is colored according to its expression level in the burn injury group compared with that of the sham group. Genes that were significantly upregulated are colored red, and those that were significantly downregulated are marked blue. The ubiquitous expression level is indicated in grey. The gene that is shown in the blank was not measured in the experiment.

In this section, we explored the parts of specific pathways involved in the pathogenesis of burn injury induction. KEGG signaling network mapping (KEGG ruo) was utilized to compare the expression levels of the sham controls and burn injury groups (Figure 5). The signaling-pathway mapping of burn-injury-induced DEGs showed that the top four pathways included the Toll-like receptor signaling pathway (Figure 5A, KEGG rno04620) involved in the 25 DEGs (Tnf, Cd80, Tlr6, Cd86, Cxcl9, Cxcl10, Tlr5, Tlr7, Traf6, Il1b, Map3k7, Ifnb1, Map2k3, Tbk1, Fos, Il12a, Tlr2, Il6, Casp8, Cd14, Nfkb1, Tlr3, Tollip, Ifna1, Mapk8, and Tlr1); the IL-17 signaling pathway (Figure 5B, KEGG rno04657), involved in 18 DEGs (Tnf, Ptgs2, Cxcl10, Traf6, Il1b, Map3k7, Traf2, Tbk1, Ccl2, Ifng, Fos, Csf3, Il6, Casp8, Tradd, Nfkb1, Csf2, Mapk8, and Cebpb); the RIG-I-like receptor signaling pathway (Figure 5C, KEGG rno04622), involved in 12 DEGs (Tnf, Cxcl10, Traf6, Map3k7, Ifnb1, Tbk1, Il12a, Casp8, Tradd, Nfkb1, Ifna1, and Mapk8); and the TNF signaling pathway (Figure 5D, KEGG rno04668), involved in the 18 DEGs (Tnf, Lta, Ptgs2, Cxcl10, Il1b, Map3k7, Ifnb1, Map2k3, Ccl2, Fos, Il6, Casp8, Tradd, Nfkb1, Csf2, Tnfrsf1a, Mapk8, and Cebpb) based on the strength value (Table S2). The top four KEGG pathways of the TLR signaling-pathway mapping of burn-injury-upregulated DEGs included the Toll-like receptor signaling pathway (Figure 5A, KEGG rno04620), involved in 18 DEGs (Tnf, Cd80, Tlr6, Cd86, Cxcl10, Tlr5, Tlr7, Il1b, Ifnb1, Fos, Tlr2, Il6, Tlr4, Casp8, Nfkb1, Tlr3, Ifna1, Mapk8, and Tlr1) and associated with the PI3K-Akt signaling pathway, apoptosis, the MyD88-dependent pathway, the NFkB signaling pathway, and the JAK-STAT signaling pathway; the IL-17 signaling pathway (Figure 5B, KEGG rno04657), involved in 15 DEGs (Tnf, Cxcl10, Il1b, Ccl2, Ifng, Fos, Csf3, Il6, Casp8, Tradd, Nfkb1, Csf2, Mapk8, and Cebpb) and associated with apoptosis, the NFkB signaling pathway, and the MAPK signaling pathway; the TNF signaling pathway (Figure 5D, KEGG rno04668), involved in the 15 DEGs (Tnf, Lta, Ptgs2, Cxcl10, Il1b, Il6, Casp8, Tradd, Nfkb1, Csf2, Mapk8, and Cebpb) and associated with the NFkB signaling pathway, ubiquitin mediated proteolysis, apoptosis, and the PI3K-Akt signaling pathway; and the RIG-I-like receptor signaling pathway (Figure 5C, KEGG rno04622), involved in eight DEGS (Tnf, Cxcl10, Ifnb1, Casp8, Tradd, Nfkb1, Ifna1, and Mapk8) and associated with ubiquitin-mediated proteolysis and the MAPK signaling pathway, based on the strength value (Table S1). The top four KEGG pathways of TLR-signaling pathway mapping of burn-injury-down-regulated DEGs included the Toll-like receptor signaling pathway (Figure 5A, KEGG rno04620), involved in seven DEGs (Traf6, Map3k7, Map2k3, Tbk1, Il12a, Cd14, and Tollip) and associated with apoptosis, the NFkB signaling pathway and the MyD88-dependent pathway; the RIG-I-like receptor signaling pathway (Figure 5C, KEGG rno04622), involved in four DEGS (Traf6, Map3k7, Tbk1, and Il12a) and associated with ubiquitin-mediated proteolysis and the MAPK signaling pathway; the NFkB signaling pathway (Figure 5D, KEGG rno04064), associated with the B cell receptor signaling pathway, the auto-ubiquitination pathway, and ubiquitin mediated proteolysis, based on the strength value (Table S3); and the IL-17 signaling pathway (Figure 5B, KEGG rno04657), involved in three DEGs (Traf6, Map3k7, and Tbk1), associated with ubiquitin-mediated proteolysis.

### 3.6. Function Analysis of Burn-Injury-Induced DEGs Related TLR Pathway

To know if the functions of DEGs have the same trend as KEGG pathway, we analyzed the functions of the DEGs (Figure 5E. Compared to the function of up-regulated DEGs, burn-injury-induced down-regulated DEGs were related to ubiquitin-conjugated enzyme-binding and cytokine binding, different from the function of up-regulated DEGs (Figure 5E). To understand the functional difference between burn-injury-induced up-regulated DEGs and down-regulated DEGs, we further analyzed DEGs involved in functional complexes. We found that the top four complexes were the I-kappaB/NF-kappaB complex, the Transcription factor ap-1 complex, the Lipopolysaccharide receptor complex, and the MHC class I peptide loading complex in up-regulated DEGs (Tables S2–S4), but the STING complex, the interleukin-1 receptor complex, the IkappaB kinase complex, and the NF-kappaB complex in down-regulated DEGs (Table S3); this results in different functions.

### 3.7. Validation of Burn-Injury-Induced TLR-Related DEGs Using qPCR

**Figure 6:**
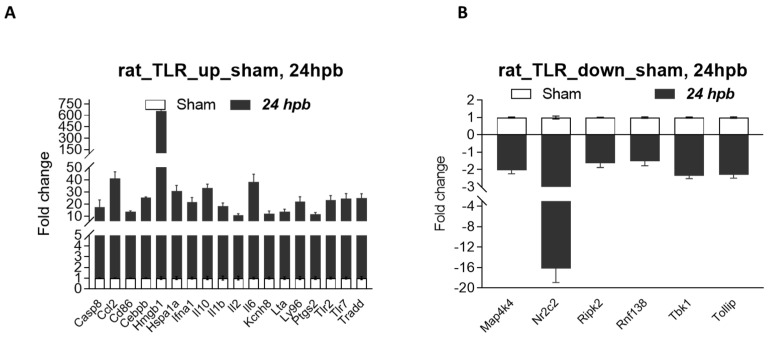
Validation of burn-induced regulated genes using qPCR. Triplicate total RNA samples from rat hearts (either sham healthy or 60% TBSA burn for 24 h) were transcribed to cDNA; then, real-time qPCR was run with our own designed primers (Table 1); (**A**) burn-injury-induced up-regulated TLR-related DEGs, and (**B**) burn-injury-induced down-regulated TLR-related DEGs.

It is known that a disadvantage of this method is that most identified differentially expressed genes/proteins are false positives once we individually test them—using qPCR for DEGs, and Western blotting/ELISA/ICH for differentially expressed proteins (DEPs). Therefore, we selected eight burn-injury-induced up-regulated genes, including Ccl2, Hmgb1, IL1a, IL10, Nfkb1, Tricam 2, Ube2n, and Ube2v1 (≥5-fold change), and all five burn-injury-induced down-regulated genes, including Map4k4, Ripk2, Rnf138, Tbk1, and Tollip (≥1.5-fold change), to be validated by qPCR. The primers for the qPCR tests are listed in Table 1. Our qPCR data show that all selected identified burn-injury-induced DEGs, whether up-regulated (Figure 6A) or down-regulated (Figure 6B), had the same trend as our microarray results.

### 3.8. Characterizing Protein–Protein Interactions through STRING Analysis

**Figure 7:**
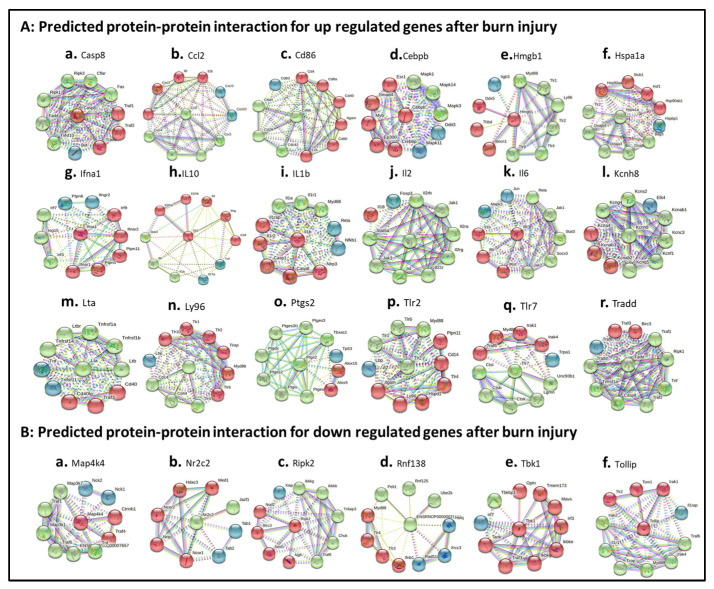
Burn-injury-induced TLR-related DEP–protein interaction networks. (**A**) burn-injury-induced up-regulated TLR-related DEP–protein interactions including Casp8 (**A.a**), Ccl2 (**A.b**), Cd86 (**A.c**), Cebpb (**A.d**), Hmgb1 (**A.e**), Hspa1a (**A.f**), Ifna1 (**A.g**), IL10 (**A.h**), IL1b (**A.i**), IL2 (**A.j**), IL6 (**A.k**), Kcnh8 (**A.l**), Lta (**A.m**), Ly96 (**A.n**), Ptgs2 (**A.o**), Tlr2 (**A.p**), Tlr7 (**A.q**), and Tradd (**A.r**) by selecting fold change ≥ 10; (**B**) burn-injury-induced down-regulated TLR-related DEP–protein interactions including Map4k4 (**B.a**), Nr2c2 (**B.b**), Ripk2 (**B.c**), Rbf138 (**B.d**), Tbk1 (**B.e**), and Tollip (**B.f**) by selecting fold change ≤ 5. The interaction network was created using STRING (Search Tool for the Retrieval of Interacting Genes/Proteins) database version 10.5. A high confidence cutoff of 0.7 or 0.9 was implemented in this work. In the resulting protein association network, proteins are presented as nodes, which are connected by lines whose thickness represents the confidence level (0.7–0.9).

String (version 11.5) was employed to postulate the protein–protein interactions and/or networks. Eighteen up-regulated DEGs (Figure 7A) and six down-regulated DEGs were analyzed (Figure 7B). For up-regulated proteins, the protein–protein interaction between 10 interacting proteins with selected DEG-derived proteins were summarized in Table S4. In detail, Casp8 directly connected with Irak1, Tlr2, Trollip and Tom1 (Figure 7A.a, red colors; Table S4). These proteins were involved in inhibits cell activation; recruits IRAK1 to the IL-1 receptor complex; inhibits IRAK1 phosphorylation and kinase activity; connects the ubiquitin pathway to autophagy. Ccl2 directly connected with CCr1, Ccr3, Ccr4, and Ccr5 (Figure 7A.b, green colors; Table S4). These proteins were involved in increasing the intracellular calcium ions level, in the control of granulocytic lineage proliferation or differentiation, and in the AKT signaling cascade. Cd86 directly interacted with Cd28, Cdc42, Ctla4 proteins (green colors in Figure 7A.c; Table S4). These proteins were involved in T-cell activation, the induction of cell proliferation and cytokine production and promotion of T-cell survival; enhances the production of IL4 and IL10 in T-cells in conjunction with TCR/CD3 ligation and CD40L co-stimulation; regulates the bipolar attachment of spindle microtubules to kinetochores, plays a role in the extension and maintenance of the formation of thin, and mediates CDC42-dependent cell migration. Cebpb directly interacted with Crebbp, Ep300, Ers1, Myb, and Smad4 (Figure 7A.d, red colors; Table S4), which are involved in the acetylates non-histone proteins, like DDX21, FBL, IRF2, MAFG, NCOA3, POLR1E/PAF53, PCNA and FOXO1, binds specifically to phosphorylated CREB and enhances its transcriptional activity toward cAMP-responsive genes, acts as a coactivator of ALX1 and as a circadian transcriptional coactivator which enhances the activity of the circadian transcriptional activators: NPAS2-ARNTL/BMAL1 and CLOCK-ARNTL/BMAL1 heterodimers (By similarity); involved in the regulation of eukaryotic gene expression and affect cellular proliferation and differentiation in target tissues, and binding to a palindromic estrogen response element (ERE) sequence or association with other DNA-binding transcription factors, such as AP-1/c-Jun, c-Fos, ATF-2, Sp1 and Sp3, to mediate ERE- independent signaling; and promotes binding of the SMAD2/SMAD4/FAST-1 complex to DNA and provides an activation function required for SMAD1 or SMAD2 to stimulate transcription. Hmgb1 directly interacted with Ddx5, Becn1, and Thbp proteins (red colors in Figure 7A.e; Table S4), which play a role in multiple membrane-trafficking pathways. Hspa1a directly interacted with Bag3, Dnaja3, Dnajb1, Dnajb4, and Tlr2 proteins (green colors in Figure 7A.f; Table S4), which play a role in protection of the proteome from stress, folding and transport of newly synthesized polypeptides, activation of proteolysis of misfolded proteins and the formation and dissociation of protein complexes, protein quality control system; inhibits HSPA1A chaperone activity and interferes with ATP binding and ubiquitination mediated by STUB1. Ifna1 directly interacted with (Figure 7A.g, red colors; Table S4), which are involved in stimulation of the production of two enzymes: a protein kinase and an oligoadenylate synthetase; act as a regulator of endoplasmic reticulum unfolded protein response, mediate dephosphorylation of EIF2AK3/PERK and regulate the EFNA5-EPHA3 signaling pathway which modulates cell reorganization and cell-cell repulsion, and regulate the hepatocyte growth factor receptor signaling pathway through dephosphorylation of MET; act downstream of various receptor and cytoplasmic protein tyrosine kinases to participate in the signal transduction from the cell surface to the nucleus, positively regulate MAPK signal transduction pathway, dephosphorylate GAB1, ARHGAP35, EGFR, CDC73 and ROCK2 resulting in stimulatation of its RhoA binding activity. IL10 directly interacted with Cd4, Ifng, Il10ra, Il10rb and Il4 proteins (Figure 7A.h, red colors; Table S4), which are involved in playing an essential role in the immune response and serves multiple functions in responses against both external and internal offenses, function primarily as a coreceptor for MHC class II molecule; have important immunoregulatory functions and antiproliferative effects on transformed cells; inhibition of the synthesis of a number of cytokines, including IFN-gamma, IL-2, IL-3, TNF and GM-CSF produced by activated macrophages and by helper T-cells; participating in at least several B-cell activation processes as well as of other cell types, co-stimulator of DNA-synthesis and enhancement of both secretion and cell surface expression of IgE and IgG1. IL1b directly interacted with Casp1, Casp8, Il1r2, Il1rap and Nlrp3 proteins (Figure 7A.i, red colors; Table S4), which are involved in a variety of inflammatory processes, important for defense against pathogens, cleaving and activating sterol regulatory element binding proteins (SREBPs), promoting apoptosis; most upstream protease of the activation cascade of caspases responsible for the TNFRSF6/FAS mediated and TNFRSF1A induced cell death; induce prostaglandin synthesis, neutrophil influx and activation, T-cell activation and cytokine production, B- cell activation and antibody production, and fibroblast proliferation and collagen production, promote Th17 differentiation of T-cells, synergize with IL12/interleukin-12 to induce IFNG synthesis from T- helper 1 (Th1) cells; modulate cellular response through non-signaling association with IL1RAP after binding to IL1B, recruit secreted IL1RAP with high affinity; play a crucial role in innate immunity and inflammation, initiate the formation of the inflammasome polymeric complex, made of NLRP3, PYCARD and CASP1 (or possibly CASP4/CASP11). IL2 directly interacted with Il21r, Il2ra, Il2rb, Il2rg, Il4, Jak1, Jak3 and Satt5a (Figure 7A.j, green colors; Table S4), which are involved in the stimulation of B-cells, monocytes, lymphokine- activated killer cells, natural killer cells, and glioma cells; regulation of immune tolerance by controlling regulatory T cells (TREGs) activity; receptor mediated endocytosis and transduces the mitogenic signals of IL2 and the stimulation of neutrophil phagocytosis by IL15; inducing the expression of class II MHC molecules on resting B-cells, enhancing both secretion and cell surface expression of IgE and IgG1 and regulations of the expression of the low affinity Fc receptor for IgE (CD23) on both lymphocytes and monocytes and IL31RA expression in macrophages; various processes such as cell growth, development, or differentiation; mediation of cellular responses to activated FGFR1, FGFR2, FGFR3 and FGFR4. IL6 directly connected with Il1b, Il6r and Il6st proteins (red colors in Figure 7A.k; Table S4), which are involved in the T-cell activation and cytokine production, B- cell activation and antibody production, and fibroblast proliferation and collagen production, promoting Th17 differentiation of T-cells. and synergizing with IL12/interleukin-12 to induce IFNG synthesis from T- helper 1 (Th1) cells; essential role in the final differentiation of B-cells into Ig- secreting cells. requirement for the generation of T(H)17 cells; activation 0f Janus kinases and mediation of signals which regulate immune response, hematopoiesis, pain control and bone metabolism. Kcnh8 directly connected with Kcnab1, Kcnc3, Kcnf1, Kcng4, Kcnq5 and Kcns2 proteins (green colors in Figure 7A.l; Table S4), which are involved in cytoplasmic potassium channel subunit that modulates the characteristics of the channel-forming alpha-subunits; an important role in the rapid repolarization of fast-firing brain neurons; formation of functional heterotetrameric channels with KCNB1 and KCNB2 and modulationof the delayed rectifier voltage- gated potassium channel activation and deactivation rates of KCNB1 and KCNB2. Lta directly connected with Ltb, Ltbr, Tnfrsf14, Tnfrsf1a and Tnfrs1b proteins (green colors in Figure 7A.m; Table S4), which are involved in the adapter molecule FADD recruits caspase-8 to the activated receptor, the resulting death-inducing signaling complex (DISC) performs caspase-8 proteolytic activation which initiates the subsequent cascade of caspases (aspartate- specific cysteine proteases) mediating apoptosis; recruiting the apoptotic suppressors BIRC2 and BIRC3 to TNFRSF1B/TNFR2. Ly96 directly connected with Cd14, Cd44, Tlr4 proteins (green colors in Figure 7A.n; Table S4), which are involved in mediating the innate immune response to bacterial lipopolysaccharide (LPS), acting as a coreceptor for TLR2:TLR6 heterodimer in response to diacylated lipopeptides and for TLR2:TLR1 heterodimer in response to triacylated lipopeptides; action via MYD88, TIRAP and TRAF6, leading to NF-kappa-B activation, cytokine secretion and the inflammatory response, LPS-independent inflammatory responses triggered by free fatty acids. Ptgs2 directly connected with Ptgds, Ptges, Ptges3, Ptges3l1, Ptgis, Ptgs2 and Tbxas1 proteins (green colors in Figure 7A.o; Table S4), which are involved in smooth muscle contraction/relaxation and a potent inhibitor of platelet aggregation, a variety of CNS functions, such as sedation, NREM sleep and PGE2-induced allodynia, and an anti-apoptotic role in oligodendrocytes; catalyzing the oxidoreduction of prostaglandin endoperoxide H2 (PGH2) to prostaglandin E2 (PGE2); being responsible for production of inflammatory prostaglandins and up-regulation of PTGS2 associated with increased cell adhesion, phenotypic changes, resistance to apoptosis and tumor angiogenesis. Tlr2 directly connected with Myd88, Tlr1 and Tlr6 proteins (green colors in Figure 7A.p; Table S4), which are involved in the Toll-like receptor and IL-1 receptor signaling pathway in the innate immune response acting via IRAK1, IRAK2, IRF7 and TRAF6, leading to NF-kappa-B activation, cytokine secretion and the inflammatory response, activating IRF1 resulting in its rapid migration into the nucleus to mediate an efficient induction of IFN-beta, NOS2/INOS, and IL12A genes. Tlr7 directly connected with Ctsb, Ctsk, Ctsl, Lgmn, and Unc93b1 proteins (green colors in Figure 7A.q; Table S4), which are involved in intracellular degradation and turnover of proteins, and in the solubilization of cross-linked TG/thyroglobulin in the thyroid follicle lumen; partially in the disorder of bone remodeling, playing an important role in extracellular matrix degradation; overall degradation of proteins in lysosomes; the regulation of cell proliferation via its role in EGFR degradation relating the processing of proteins for MHC class II antigen presentation in the lysosomal/endosomal system. Tradd directly connected with Casp8, Fadd, Ripk1, Tnf and Tnfrsf1a proteins (green colors in Figure 7A.r; Table S4), which are involved in being responsible for the TNFRSF6/FAS mediated and TNFRSF1A induced cell death; interferon-mediated antiviral immune response, playing a role in the positive regulation of interferon signaling; being potent pyrogen causing fever by direct action or by stimulation of interleukin-1 secretion; the resulting death-inducing signaling complex (DISC) performing caspase-8 proteolytic activation which initiates the subsequent cascade of caspases (aspartate- specific cysteine proteases) mediating apoptosis.

*For down-regulated EDGs,* Map4k4 directly interacted with Ctnnb1 and Traf4 (red colors in Figure 7B.a; Table S5), which are involved in acting as a coactivator for transcription factors of the TCF/LEF family, leading to the activation of Wnt-responsive genes and the regulation of cell adhesion. Nr2c2 directly interacted with Jazf1 (green colors in Figure 7B.b; Table S5), which are involved in acting as a repressor or activator of transcription, being as an important repressor of nuclear receptor signaling pathways such as retinoic acid receptor, retinoid X, vitamin D3 receptor, thyroid hormone receptor and estrogen receptor pathways, regulating gene expression during the late phase of spermatogenesis and activating transcriptional activity of LHCG. Ripk2 directly interacted with Birc3, Ngfr, Nod1 and Nod2 (red colors in Figure 7B.c; Table S5), which are involved in forming a heterodimeric receptor with SORCS2 that binds the precursor forms of NGF, BDNF and NTF3 with high affinity, and has much lower affinity for mature NGF and BDNF; an essential role in modulation of innate and adaptive immune responses and upon stimulation by bacterial peptidoglycans, NOD1 and NOD2 are activated, oligomerize and recruit RIPK2 through CARD-CARD domains. Rnf138 directly interacted with ENSRNOP00000021061, Peli1, Rnf125 and Ube2k proteins (green colors in Figure 7B.d; Table S5), which are involved in protein ubiquitarians. Tbk1 directly interacted with Ikbke, Ikekg, Irf3, Mavs, Optn, Tank, Tbk1, Tmem173 and Traf3 proteins (red colors in Figure 7B.e; Table S5), which are involved in regulatory subunit of the IKK core complex which phosphorylates inhibitors of NF-kappa-B thus leading to the dissociation of the inhibitor/NF-kappa-B complex and ultimately the degradation of the inhibitor; acting downstream of DDX58/RIG-I and IFIH1/MDA5, which detect intracellular dsRNA produced during viral replication, to coordinate pathways leading to the activation of NF-kappa-B, IRF3 and IRF7, and to the subsequent induction of antiviral cytokines such as IFN-beta and RANTES (CCL5); the maintenance of the Golgi complex, in membrane trafficking, in exocytosis, through its interaction with myosin VI and Rab8; being innate immune response triggered in response to non-CpG double- stranded DNA from viruses and bacteria delivered to the cytoplasm, acting by recognizing and binding cyclic di-GMP (c-di-GMP), a second messenger produced by bacteria, and cyclic GMP-AMP (cGAMP), a messenger produced in response to DNA virus in the cytosol: upon binding of c-di-GMP or cGAMP. Tollip directly interacted with Irak1, Tlr2 and Tom1 proteins (red colors in Figure 7B.f; Table S5), which are involved in connection of the ubiquitin pathway to autophagy by functioning as a ubiquitin-ATG8 family adapter and thus mediating autophagic clearance of ubiquitin conjugates. The TOLLIP-dependent selective autophagy pathway plays an important role in clearance of cytotoxic polyQ proteins aggregates.

## 4. Discussion

The analysis and identification of differentially expressed genes remains difficult to accomplish. With microarray technology, a sensitive and reliable technique has emerged. Microarrays were utilized on burn injury rats to help develop arrays focused on the genes relevant to the cardiac response in burn injury. The Rat TLR RT2 Profiler^TM^ PCR Array was used in this study, allowing us to identify genes known in burn-injury-induced cardiac inflammation.

Recently, Keyloun, J.W. et al. [22] published an excellent, extensive study on severe burn associated with immune pathways, using the transcriptome response in whole rat blood; this involved the inflammatory response as well as differentially regulated genes and immune pathways. This study aimed to characterize longitudinal changes in the cardiac TLR-related genes during the first 24 h after burn injury, and to compare the TLR-signaling pathway response between rats with and without severe burns. In our study, we focused on the identification of TLR-related genes specific to the cardiac response to burn injury using the Qiagen Rat TLR RT2 Profiler^TM^ PCR Array. A total of 45 up-regulated genes and 5 down-regulated genes were identified. A total of 8 up-regulated DEGs and 5 down-regulated DEGS after burn injury were validated using qPCR. Further studies showed that severe burn injury is associated with TLR-like response signaling, RIG-I-like receptor signaling, the IL-17 signaling pathway and the NFkB signaling pathway through utilization of KEGG. Functional analysis demonstrated that the DEGs were linked to type I IFN receptor binding, IL-1 receptor binding, JUN kinase activity binding, and death receptor activity binding.

The immune response that follows a burn injury remains a topic of interest. The innate immune system causes initial systemic inflammation, followed by a compensatory anti-inflammatory response. This is evidenced by the suppression of the adaptive immune system [23,24]. Burn injury is also associated with a sustained cytokine response that characterizes the injury as a chronic condition [25,26]. Patients are known to have long-term immune suppression as a result of this [27]. The data presented here suggest that dysregulated immune function in rats with severe burns may begin as early as the first 24 h after injury. Among the enriched pathways, the TLR4/NF-kappaB signaling pathway in burn-induced intestinal inflammation and oxidative stress is particularly important [28].

Damage-associated molecular patterns (DAMPs) and pathogen-associated molecular patterns (PAMPs) are released from burn-injured tissue and enter systemic circulation. This creates a downstream cascade that activates pattern-recognition receptors (PRR), including TLRs, to stimulate cytokine secretion. This causes dysregulation and elevation of systemic cytokine levels, a dysfunctional immune system, increased risks of infection, decreased wound healing, and excessive scarring in burn patients [29]. During the first 2 days following a partial-thickness burn injury, the zone of necrosis can expand during the resuscitation. This burn wound progression is thought to result from progressive ischemia. Contributing factors include increased capillary permeability, tissue hypoperfusion, oxidative stress, and capillary permeability. These factors are exacerbated by locally released signaling molecules from the extracellular matrix and ruptured necrotic cells (i.e., DAMPs and PAMPs) that activate inflammatory mediator production in nearby cells [30]. Knowledge of these pathways is pivotal to developing treatments that ameliorate local and systemic inflammation. This may be necessary to prevent long-term sequela, including cardiac dysfunction. The TLR-signaling pathways sense damaged tissue and are involved in wound repair, but also contribute to burn wound progression and systemic inflammation [29]. Early after burn injury, DAMPs and cytokines increase in the circulation, and contribute to systemic inflammation and secondary tissue damage. These increase susceptibility to infection, impair healing, and worsen scarring in these injuries. Signaling may act as a therapeutic target for improving burn outcomes. This study identified 50 DEGs following burn injury in 84 rat TLR-signaling-associated genes in 96 wells, indicating that TLR-signaling genes were the top targets for cardiac response to burn injury. They play a role in the damage sustained by cardiac myocytes.

Burn injury progression also involves ischemia/reperfusion (I/R) injury. Blood flow in burned tissue fluctuated repetitively (Laser Doppler Imaging) in parallel to changes in base deficit on the day of injury [31,32]. In other types of tissue damage with I/R injury, the importance of TLR signaling, including HMGB1-TLR signaling, has been demonstrated in a large number of in vivo studies [32]. In peri-burn tissue during injury progression, necrotic cells released HMGB1 from chromatin into the cytoplasm and extracellular space [33]. Then, this activates TLR4 to induce cytokines [34], resulting in inflammation [35]. HMGB1-TLR signaling has not been studied in burn-injured heart tissue. Our data show similar results in that burn injury was involved in more highly expressed TLR 2, TLR 4, and HMGB1 in cardiac tissue. This suggests that HMGB1-TLRs signaling can mediate heart tissue damage under cardiac redox conditions, as occurs during wound progression [9,11] and that HMGB1 may be generally required for recruiting neutrophils to necrotic tissue, where they may amplify tissue injury. This would urge us to further study the roles of HMGB1-TLR signaling in the cardiac response to burn injury in vivo and in vitro.

Cardiomyocyte secretion of multiple cytokines including TNF-α, IL-1β, and IL-6 after burn injury is higher in myocardial tissue than in systemic circulation [2]. In this study, we show significant increases in inflammatory mediators such as IL-1β, IL-6, IL10, and Ccl2 in cardiac tissue following burn injury, similar to the results from myocyte supernatants after burn injury [36,37]. Thus, these data suggest that higher cytokine levels measured in heart tissue after burn injury are, indeed, myocyte-derived cytokines [38]. Though the mechanisms by which burn injury alters cardiac contractile function remain unknown, our current data strengthen the evidence of cardiomyocyte secretion of inflammatory cytokines on the heart after burn injury [39,40].

The CCL2/CCR2 axis regulates the inflammatory recruitment of classical monocytes in atherosclerosis, as well as in the infarcted heart [41,42]. CCL2 is associated with the severity of heart failure, and was found to be expressed in high levels in early burn injury [43]., The timing of this expression coincides with the increased presence of myocytes [44]. In mice, serum CCL2 is found to be significantly higher on day 1 post-burn-injury when compared to sham controls [24]. This study shows a significant increase in CCL2 in 60% TBSA rats, suggesting that CCL2 plays a role in burn-induced heart inflammation.

High-mobility group box 1 (Hmgb1) serves as an inflammation driver and mediator of cardiovascular disease [45]. HMGB1 binds to different receptors (e.g., RAGE, TLR4, and TLR2) on neighboring cells and activates signaling pathways that lead to inflammation, apoptosis, autophagy, necrosis, fibrosis, and cardiac dysfunction [45]. HMGB1 is involved in a wide range of cardiovascular pathophysiology and participates in hypertrophy, myocardial infarction, and remodeling; this subsequently leads to heart failure and pulmonary hypertension [46,47]. HMGB1/IL-1β complexes released after burn injuries modulate immune responses. These immune responses may be mediated my microvesicles, and these complexes might serve as novel immune targets for the treatment of systemic immune responses in burn injury [48], whose process might be largely dependent on the TLR-dependent MAPK/NF-κB signal pathway [49]. This study revealed that burn injury significantly increased cardiac Hmgb1, TLR2, TLR4, and IL1, providing more evidence for the described observations.

IL-1 exerts pro-apoptotic and hypertrophic effects on cardiomyocytes, while depressing cardiac contractility through several different pathways [50,51]. This pathway represents yet another potential therapeutic target. IL-1β independently impaired cardiac myocyte viability and produced cardiac contractility abnormalities following injury [52]. IL-10 is significantly increased in the serum within 6 h following myocardial ischemia/reperfusion injury [53]. Baseline circulating levels of the anti-inflammatory IL-10 are positively associated with risk of cardiovascular disease [54]. IL-10 plays key roles in the progression of cardiac fibrosis [55], which can result in the cardiac injury described. IL-10 is known to suppress the synthesis of proinflammatory cytokines, and their expression was significantly increased after burn injury, as seen in our data [56,57]. The roles and potential therapeutic use for IL1 and IL10 in cardiac inflammation after burn injury warrants further investigation.

Transcription factor NFκB is a key player in the development and progression of both inflammation and cardiovascular damage [58]. NFκB is a ubiquitous transcription factor involved in cellular responses to stimuli such as stress, cytokines, free radicals, ultraviolet irradiation, oxidized LDL, burn injury, and bacterial or viral antigens [59,60]. NFκB is activated in the heart in many conditions: during acute ischemia and reperfusion injury, during unstable angina, or in response to preconditioning [61]. Based on the published literature and our work, it is worth further investigating its mechanisms and potential therapeutic capacity in burn-injury-induced heart dysfunction.

Ube2v1 is a mammalian homolog of yeast MMS2 and a cofactor of ubiquitin-conjugating enzyme E2 N (Ube2n) [62,63]. Ube2v1 is involved in protein aggregate formation, at least in part, by enhancing K63 ubiquitination in response to a proteotoxic stimulus. Ube2v1 modulates the ubiquitin-proteasome system performance separately from autophagy. Ube2v1 deficiency improves cardiac performance with increased lifespan in vivo [62,64]. To perform this task, it must form complexes with Ube2n [65]. Our results show that both Ube2v1 and Ube2n gene expression after burn injury was increased compared to the sham control, indicating that the Ube2v1–Ube2n complex is also targeted and worthy of further study.

Mitogen-activated protein kinase kinase kinase kinase-4 (MAP4K4) is an upstream member of the MAPK superfamily, and is implicated in human cardiac-muscle-cell death from oxidative stress. A role of MAP4K4 was seen in cardiomyocytes in doxorubicin cardiomyopathy [66,67]. MAP4K4 is now appreciated as a pivotal mediator of inflammation, cytoskeletal function, and, notably, cell death, with well-established contributions to cancer, diabetes, and neurodegeneration [68,69]. MAP4K4 function in the heart is theoretical, but a pathobiological role is suggested by its engagement in transforming-growth-factor-b-activated kinase-1 (TAK1/MAP3K7), JNK, and p38 MAPK [70]. Very interestingly, MAP4K4 was significantly decreased after burn injury, seemingly the opposite of other kinds of stress-induced heart dysfunction. This warrants further study, to discover how the mechanism differs in burn injury.

Receptor-interacting serine-threonine protein kinase 2 (Ripk2) is involved in the signaling pathways of members of the NOD family of cytosolic pattern-recognition receptors. Ripk2 has been implicated in the caspase-1 pathway of hypoxia and ischemia-induced neuronal cell death, as well as the pathology of neuroinflammatory diseases such as multiple sclerosis. However, despite being seen in other ischemia/reperfusion injuries, it has not been previously described in the context of cardiac injury. RIPK2 mediates inflammatory signaling through the bacteria-sensing receptors NOD1 and NOD2 [71]. Stimulation of NOD2 recruits RIPK2 and ubiquitin (Ub) ligases, including IAP (inhibitor of apoptosis) proteins and LUBAC (linear ubiquitin chain assembly complex) [72,73,74]. RIPK2 kinase activity is necessary for the NOD2 inflammatory signaling pathway [71]. No articles reported relationships between Ripk2 and heart function in our literature review. Our results showed a significant decrease in Ripk2 gene expression after burn injury, and were the first to identify the DEG of Ripk2 after burn injury.

RNF138 has been shown to function as an E3 ligase in the regulation of Wnt-β-catenin signaling by interacting with the negative regulator NLK, to target the Wnt-activating cofactor TCF/LEF for degradation by ubiquitination [75,76,77]. Our previous work found burn-induced heart dysfunction to be associated with cardiac apoptosis (Wen, 2020 #5221; Wen, 2020 #5222), this study suggests that burn-induced heart apoptosis was related to Rnf138 deficiency.

TBK1 was decreased in MI/R injury in mice [78]. In addition to innate immunity, TBK1 plays a critical role in cardiac diseases [79]. TBK1 overexpression improved cardiac function, infarct size and cardiomyocyte apoptosis impaired by ischemia/reperfusion injury. It also prevented the disruption of mitochondria and cardiac muscle fibers in this injury [78]. Whether TBK1 overexpression would improve burn-induced heart dysfunction will be investigated in our further proposed projects.

Tollip, a well-established endogenous modulator of Toll-like receptor signaling, is involved in cardiovascular diseases [80], including cardiac remodeling after chronic pressure overload and myocardial infarction [81,82]. Tollip can physically associate with TLR2 and TLR4 to suppress TLR signaling [83] and is a widely accepted regulator of TLR signaling in the immune response [80]. The mechanism of Tollip decrease following burn injury will be further investigated. The Tollip gene was decreased, but its tightly associated genes, including TLR2 and TLR4, were increased in our data.

For human burn patients, burn injury has a profound effect on the immune system and immune cells. Inflammation begins immediately after burn injury and lasts for weeks to months, depending on the severity of the burn injury [84]. We recognize endogenous factors, such as damage-associated molecular patterns (DAMPs) or alarmins, that are generated as a result of burn-mediated tissue damage, including heart tissue [85,86,87,88,89]. The response begins with the release of histamine, free radicals, and inflammatory cytokines, which induce vasodilation and tissue edema, including heart tissue [89,90]. Burn injury also depresses cardiac function within a few hours of injury, lasting ~24–48 h, via oxidative stress; the release of inflammatory mediators (such as IL-6 and tumor necrosis factor (TNF)); and cellular alterations (such as apoptosis and necrosis) [85,91,92,93]. Major burns are accompanied by a strong inflammatory response, which will most often lead to systemic response inflammatory syndrome, followed by sepsis; finally, it will induce multiple-organ failure [94]. Immediately after a burn, cardiomyopathy, along with dendritic cells (DCs) and macrophages, is involved in the synthesis of numerous immune cells within the innate immunity, such as cytokines, chemokines, and adipocytokines, as well as catecholamines, cortisol, and reactive oxygen species (ROS); these mediate both the local inflammatory response and systemic inflammation [40,95,96]. The inflammatory process plays a key role in the healing of burn injuries. Another important finding, at one hour post-burn, was early synthesis of the Th1 pro-inflammatory cytokines (TNF-α and IFN-γ), which play a role in activating pro-inflammatory macrophages MIP-1α, MIP-1β, respectively [97]. Proper management goals are represented by the decrease in the inflammatory phase [98]. For the rat burn model, it showed that early burn (1 h after burn injury) caused inflammatory responses [99], and that the administration of anti-TNF-α antibodies and anti-IL-1β, respectively, resulted in a reduction in necrosis extension in the burn wounds of rats [100,101,102]. Taken together, this suggests that immunotherapy to protect the heart should be administered immediately during the early phase after a burn injury, in either human patients or animal models.

## 5. Conclusions

The clinical relevance of the published reports and our data support the fact that myocardial injury and dysfunction occur in several injury and disease states, including burn injury [4,38]. A better understanding of the cell signaling mechanisms by which an extracellular stimulus, such as burn injury, triggers a cellular cascade that produces cytokine secretion and organ dysfunction will allow the development of therapeutic strategies that support and maintain cardiac function. Our studies indicate that TLR-signaling genes are the top targets for the cardiac response to burn injury, and play a very important role in the damage of cardiac myocytes; moreover, TLR signaling may potentially act as a therapeutic target for improving burn outcomes.

## Figures and Tables

**Table 1:** Oligonucleotides used for validation of the identified burn-induced TLR gene expressions.

Gene Expressions.	5′-Forward-3′	5′-Reverse-3′	Amplicon Size (bp)	Accession #
Casp8	AATAAAGACAACCCGAGGAACA	ATGCCACAGCCCATCTTCACACTA	101	NM_022277.1
Ccl2	GTCTCAGCCAGATGCAGTTAAT	CTGCTGGTGATTCTCTTGTAGTT	105	NM_031530.1
Cd86	CCGAGTGAGCTCGTAGTATTT	GGTACTTGGCATTCACGTTATC	100	NM_020081.1
Cebpb	CTTGATGCAATCCGGATCAAAC	CCCGCAGGAACATCTTTAAGT	113	NM_024125.5
Hmgb1	TCGGCCTTCTTCTTGTTCTG	GTTGTTCCACATCTCTCCTAGTT	108	NM_012963.2
Hspa1a	CGTTTGACACTCTGTTGCTTTC	ACGGCCAGGCAAGATTATAC	89	NM_031971.2
Ifna	AGAGAGAGAGAGAGAGAGAGAGA	GTGTGATTCCACATTTGCAGTAG	110	XM_001076062.4
IL1b	GAGGCCATAGCCCATGATTTA	CTCCTGCTTGACGATCCTTATC	105	NM_017019.2
IL10	AGTGGAGCAGGTGAAGAATG	GAGTGTCACGTAGGCTTCTATG	109	NM_012854.2
IL2	GCAGGCCACAGAATTGAAAC	CCAGCGTCTTCCAAGTGAA	108	NM_053836.1
IL6	GAAGTTAGAGTCACAGAAGGAGTG	GTTTGCCGAGTAGACCTCATAG	105	M26744.1
Kcnh8	GAAGACAGAGCCAAAGGAAGA	CAGGTGTCCTGAGATGTGATAAA	96	NM_145095.2
Lta	GCGTCAGTTACCACAGAACA	GCCTCGGGCTTTCTTCTAAA	100	NM_080769.2
Ly98	CCGAAGCGCAAGGAAATTG	TGTGATGGCCCTTAGGAAATAG	133	NM_001024279.1
Map4k4	CTGGTGGAAGTGGTTGGAAA	CATCCTCGGTGACATCCATAAC	97	NM_001106904.1
Nr2c2	CTGATAGCCACTCCCACATTT	GAACTGTACCATCCTCACGTATC	118	NM_017323.2
Ptgs2	GGCCATGGAGTGGACTTAAA	GTCTTTGACTGTGGGAGGATAC	132	NM_017232.3
Ripk2	CCTTTGCCTCCTGTCTTTCT	CCTCTACCCAACCAGCATATT	100	NM_001191865.1
Rnf 138	TTACCAGCCTCTGTTTCACTAC	TGACTTGGCCTTCTCTACATTAC	83	NM_053588.3
Tbk1	GAAGAAGCTGAAGGAGGAGATG	CAGACTCCCGAAGAAGCTAAAG	137	NM_001106786.1
Tlr2	CCAAGAGGAAGCCCAAGAAA	CATGAGGTTCTCCACCCAATAG	98	NM_198769.2
Tlr7	AACCTTTCCCAGAGCATACAG	AGCCTCTGATGGGACAAATAAA	110	NM_001097582.1
Tollip	CCCTCCTCTGATGTTGTATGTG	GGTAAGCAAGACAGGAGGTTAG	108	NM_001109668.1
Tradd	CTGGACCTTCTGAAACCTAGATG	CTTAGGTCCCACGATGAGAATG	106	NM_001100480.1

**Table 2:** Rat TLR RT2 Profiler PCR Array (Qiagen). NCBI access number, abbreviation (symbol) and full name/function (description) of the 84 TLR-related genes analyzed using the PCR array. The column “fold change” indicates the tested gene expression in burn injury group vs. sham control.

Position	Refseq	Symbol	Description	Fold Change
A01	NM_001007798	Btk	Bruton agammaglobulinemia tyrosine kinase	1.05
A02	NM_022277	Casp8	Caspase 8	2.12
A03	NM_031530	Ccl2	Chemokine (C-C motif) ligand 2	43.61
A04	NM_021744	Cd14	CD14 molecule	1.23
A05	NM_001106405	Cd180	CD180 molecule	1.05
A06	NM_012926	Cd80	Cd80 molecule	1.05
A07	NM_020081	Cd86	CD86 molecule	1.08
A08	NM_024125	Cebpb	CCAAT/enhancer binding protein (C/EBP), beta	2.03
A09	NM_001107588	Chuk	Conserved helix–loop–helix ubiquitous kinase	1.55
A10	NM_001005897	Clec4e	C-type lectin domain family 4, member e	1.05
A11	NM_053852	Csf2	Colony stimulating factor 2 (granulocyte-macrophage)	1.05
A12	NM_017104	Csf3	Colony stimulating factor 3 (granulocyte)	1.05
B01	NM_139089	Cxcl10	Chemokine (C-X-C motif) ligand 10	1.51
B02	NM_019335	Eif2ak2	Eukaryotic translation initiation factor 2-alpha kinase 2	2.05
B03	NM_152937	Fadd	Fas (TNFRSF6)-associated via death domain	2.00
B04	NM_022197	Fos	FBJ osteosarcoma oncogene	4.18
B05	NM_012963	Hmgb1	High-mobility group box 1	54.92
B06	NM_001098241	Hras	Harvey rat sarcoma virus oncogene	2.16
B07	NM_031971	Hspa1a	Heat shock 70 kD protein 1A	2.47
B08	NM_022229	Hspd1	Heat shock protein 1 (chaperonin)	4.07
B09	NM_001014786	Ifna1	Interferon-alpha 1	1.74
B10	NM_019127	Ifnb1	Interferon beta 1, fibroblast	1.05
B11	NM_138880	Ifng	Interferon gamma	1.05
B12	NM_053355	Ikbkb	Inhibitor of kappa light polypeptide gene enhancer in B-cells, kinase beta	1.42
C01	NM_012854	Il10	Interleukin 10	5.49
C02	NM_053390	Il12a	Interleukin 12a	1.03
C03	NM_017019	Il1a	Interleukin 1 alpha	11,883,604.11
C04	NM_031512	Il1b	gb Interleukin 1 beta	1.47
C05	NM_013123	Il1r1	Interleukin 1 receptor, type I	−1.13
C06	NM_053836	Il2	Interleukin 2	1.49
C07	NM_012589	Il6	Interleukin 6	3.12
C08	NM_017020	Il6r	Interleukin 6 receptor	1.65
C09	NM_001127555	Irak1	Interleukin-1-receptor-associated kinase 1	4.64
C10	NM_001025422	Irak2	Interleukin-1-receptor-associated kinase 2	1.06
C11	NM_012591	Irf1	Interferon regulatory factor 1	3.11
C12	NM_001006969	Irf3	Interferon regulatory factor 3	2.66
D01	NM_021835	Jun	Jun oncogene	1.62
D02	NM_145095	Kcnh8	Potassium voltage-gated channel, subfamily H (eag-related), member 8	1.09
D03	NM_080769	Lta	Lymphotoxin alpha (TNF superfamily, member 1)	1.08
D04	NM_001024279	Ly96	Lymphocyte antigen 96	1.76
D05	NM_012798	Mal	Mal, T-cell differentiation protein	1.81
D06	NM_001100674	Map2k3	Mitogen-activated protein kinase kinase 3	−1.43
D07	NM_001030023	Map2k4	Mitogen-activated protein kinase kinase 4	2.75
D08	NM_053887	Map3k1	Mitogen-activated protein kinase kinase kinase 1	1.08
D09	NM_001107920	Map3k7	Mitogen-activated protein kinase kinase kinase 7	−1.05
D10	NM_001106904	Map4k4	Mitogen-activated protein kinase kinase kinase kinase 4	−2.04
D11	NM_053829	Mapk8	Mitogen-activated protein kinase 8	2.32
D12	NM_001100673	Mapk8ip3	Mitogen-activated protein kinase 8 interacting protein 3	1.74
E01	NM_017322	Mapk9	Mitogen-activated protein kinase 9	1.71
E02	NM_198130	Myd88	Myeloid differentiation primary response gene 88	2.10
E03	NM_001276711	Nfkb1	Nuclear factor of kappa light polypeptide gene enhancer in B-cells 1	5.52
E04	NM_001008349	Nfkb2	Nuclear factor of kappa light polypeptide gene enhancer in B-cells 2, p49/p100	1.25
E05	NM_001105720	Nfkbia	Nuclear factor of kappa light polypeptide gene enhancer in B-cells inhibitor, alpha	2.12
E06	NM_030867	Nfkbib	Nuclear factor of kappa light polypeptide gene enhancer in B-cells inhibitor, beta	1.54
E07	NM_212509	Nfkbil1	Nuclear factor of kappa light polypeptide gene enhancer in B-cells inhibitor-like 1	3.40
E08	NM_001108133	Nfrkb	Nuclear factor related to kappaB binding protein	−1.19
E09	NM_017323	Nr2c2	Nuclear receptor subfamily 2, group C, member 2	1.00
E10	NM_001100565	Peli1	Pellino 1	4.12
E11	NM_053373	Pglyrp1	Peptidoglycan recognition protein 1	2.17
E12	NM_013196	Ppara	Peroxisome proliferator activated receptor alpha	−1.20
F01	NM_017232	Ptgs2	Prostaglandin-endoperoxide synthase 2	1.05
F02	XM_006221869	Rel	V-rel avian reticuloendotheliosis viral oncogene homolog	1.07
F03	NM_199267	Rela	V-rel reticuloendotheliosis viral oncogene homolog A (avian)	1.96
F04	NM_001191865	Ripk2	Receptor-interacting serine-threonine kinase 2	−1.63
F05	NM_053588	Rnf138	Ring finger protein 138	−1.53
F06	NM_001105817	Sarm1	Sterile alpha and TIR motif containing 1	3.57
F07	NM_001106786	Tbk1	TANK-binding kinase 1	−2.36
F08	NM_001108890	Ticam2	Toll-like receptor adaptor molecule 2	11,993,941.01
F09	NM_001172120	Tlr1	Toll-like receptor 1	1.05
F10	NM_198769	Tlr2	Toll-like receptor 2	1.87
F11	NM_198791	Tlr3	Toll-like receptor 3	1.05
F12	NM_019178	Tlr4	Toll-like receptor 4	2.03
G01	NM_001145828	Tlr5	Toll-like receptor 5	1.05
G02	NM_207604	Tlr6	Toll-like receptor 6	1.48
G03	NM_001097582	Tlr7	Toll-like receptor 7	1.96
G04	NM_198131	Tlr9	Toll-like receptor 9	1.61
G05	NM_012675	Tnf	Tumor necrosis factor (TNF superfamily, member 2)	1.05
G06	NM_013091	Tnfrsf1a	Tumor necrosis factor receptor superfamily, member 1a	−1.15
G07	NM_001024771	Tnip2	TNFAIP3 interacting protein 2	2.10
G08	NM_001109668	Tollip	Toll interacting protein	−2.29
G09	NM_001100480	Tradd	TNFRSF1A-associated via death domain	2.02
G10	NM_001107754	Traf6	Tnf-receptor-associated factor 6	−1.09
G11	NM_053928	Ube2n	Ubiquitin-conjugating enzyme E2N (UBC13 homolog, yeast)	6.17
G12	NM_001110345	Ube2v1	Ubiquitin-conjugating enzyme E2 variant 1	5.39

**Table 3:** List of DEGs of TLR-signaling pathways between burn-injury and sham rats.

Position		Symbol	Description	Fold Regulation (24 hpb vs. Sham)
	Toll-like receptor			
F10		Tlr2	Toll-like receptor 2	1.87
F12		Tlr4	Toll-like receptor 4	2.03
G03		Tlr7	Toll-like receptor 7	1.96
G04		Tlr9	Toll-like receptor 9	1.61
	Effectors			
C09		Irak1	Interleukin-1 receptor-associated kinase 1	4.64
	Interacting proteins and adaptors			
B07		Hspa1a	Heat shock 70 kD protein 1A	2.47
D12		Mapk8ip3	Mitogen-activated protein kinase 8 interacting protein 3	1.74
G08		Tollip	Toll interacting protein	−2.29
	Apoptosis			
A02		Casp8	Caspase 8	2.12
	Ubiquitin-conjugating pathway			
E10		Peli1	Pellino 1	4.12
G11		Ube2n	Ubiquitin-conjugating enzyme E2N (UBC13 homolog, yeast)	6.17
G12		Ube2v1	Ubiquitin-conjugating enzyme E2 variant 1	5.39
A03		Ccl2	Chemokine (C-C motif) ligand 2	43.61
A08		Cebpb	CCAAT/enhancer binding protein (C/EBP), beta	2.03
A09		Chuk	Conserved helix–loop–helix ubiquitous kinase	1.55
B02		Eif2ak2	Eukaryotic translation initiation factor 2-alpha kinase 2	2.05
B04		Fos	FBJ osteosarcoma oncogene	4.18
B05		Hmgb1	High-mobility group box 1	54.92
B08		Hspd1	Heat shock protein 1 (chaperonin)	4.07
	Regulation of adaptive immunity			
C01		Il10	Interleukin 10	5.49
E03		Nfkb1	Nuclear factor of kappa light polypeptide gene enhancer in B-cells 1	5.52
E06		Nfkbib	Nuclear factor of kappa light polypeptide gene enhancer in B-cells inhibitor, beta	1.54
	Downstream pathway of Toll-like receptors NFKB signaling			
C01		Il10	Interleukin 10	5.49
C09		Irak1	Interleukin-1-receptor-associated kinase 1	4.64
D10		Map4k4	Mitogen-activated protein kinase kinase kinase kinase 4	−2.04
E03		Nfkb1	Nuclear factor of kappa light polypeptide gene enhancer in B-cells 1	5.52
E06		Nfkbib	Nuclear factor of kappa light polypeptide gene enhancer in B-cells inhibitor, beta	1.54
	JNK/p38 signaling			
D12		Mapk8ip3	Mitogen-activated protein kinase 8 interacting protein 3	1.74
	JAK/STAT signaling			
A03		Ccl2	Chemokine (C-C motif) ligand 2	43.61
F07		Tbk1	TANK-binding kinase 1	−2.36
	Interferon regulatory factor signaling			
B01		Cxcl10	Chemokine (C-X-C motif) ligand 10	1.51
C11		Irf1	Interferon regulatory factor 1	3.11
F07		Tbk1	TANK-binding kinase 1	−2.36
	Cytokine signaling			
A03		Ccl2	Chemokine (C-C motif) ligand 2	43.61
C09		Irak1	Interleukin-1-receptor-associated kinase 1	4.64
	NFkB/IL6 pathway			
B09		Ifna1	Interferon-alpha 1	1.74
C03		Il1a	Interleukin 1 alpha	11,883,604.11
C07		Il6	Interleukin 6	3.12
	JUN-MARK signaling			
D01		Jun	Jun oncogene	1.62
D04		Ly96	Lymphocyte antigen 96	1.76
D11		Mapk8	Mitogen-activated protein kinase 8	2.32
E01		Mapk9	Mitogen-activated protein kinase 9	1.71
	Others			
E11		Pglyrp1	Peptidoglycan recognition protein 1	2.17
F04		Ripk2	Receptor-interacting serine-threonine kinase 2	−1.63
F05		Rnf138	Ring finger protein 138	−1.53
F06		Sarm1	Sterile alpha and TIR motif containing 1	3.57
F08		Ticam2	Toll-like receptor adaptor molecule 2	11,993,941.01
G09		Tradd	TNFRSF1A-associated via death domain	2.02

**Table 4:** Top 10 Enriched GO terms of the DEGs.

Sub Ontologies	ID	Description	Observed Gene Count	Bachground Gene Count	Strength
GO-MF	GO:0035663	Toll-like receptor 2 binding	2	3	2.41
GO-MF	GO:0071723	Lipopeptide binding	3	5	2.36
GO-MF	GO:0035325	Toll-like receptor binding	4	14	2.04
GO-MF	GO:0050135	NAD(P)+ nucleosidase activity	5	18	2.03
GO-MF	GO:0061809	NAD+ nucleotidase, cyclic ADP-ribose generating	5	18	2.03
GO-MF	GO:0005132	Type i interferon receptor binding	2	10	1.89
GO-MF	GO:0038187	Pattern recognition receptor activity	4	21	1.87
GO-MF	GO:0005149	interleukin-1 receptor binding	3	17	1.83
GO-MF	GO:0005164	Tumor necrosis factor receptor binding	5	34	1.75
GO-MF	GO:0035370	UBC13-UEV1A complex	2	4	2.29
GO-CC	GO:0033256	I-kappaB/NF-kappaB complex	3	9	2.11
GO-CC	GO:0046696	Lipopolysaccharide receptor complex	2	8	1.98
GO-CC	GO:0045335	Phagocytic vesicle	4	135	1.06
GO-CC	GO:0042025	Host cell nucleus	5	187	1.01
GO-CC	GO:0030139	Endocytic vesicle	5	218	0.95
GO-CC	GO:0045121	Membrane raft	10	446	0.94
GO-CC	GO:0043235	Receptor complex	9	482	0.86
GO-CC	GO:0009897	External side of plasma membrane	8	512	0.78
GO-CC	GO:0098552	Side of membrane	9	730	0.68
GO-CC	GO:0005615	Extracellular space	21	1776	0.66
GO-BP	GO:0002874	Regulation of chronic inflammatory response to antigenic stimulus	3	3	2.59
GO-BP	GO:0060559	Positive regulation of calcidiol 1-monooxygenase activity	3	3	2.59
GO-BP	GO:0002876	Positive regulation of chronic inflammatory response to antigenic stimulus	2	2	2.59
GO-BP	GO:0070340	Detection of bacterial lipopeptide	2	2	2.59
GO-BP	GO:0071727	Cellular response to triacyl bacterial lipopeptide	2	2	2.59
GO-BP	GO:0035711	T-helper 1 cell activation	3	4	2.46
GO-BP	GO:0001781	Neutrophil apoptotic process	2	3	2.41
GO-BP	GO:0060558	Regulation of calcidiol 1-monooxygenase activity	4	8	2.29
GO-BP	GO:0071221	Cellular response to bacterial lipopeptide	4	8	2.29
GO-BP	GO:0071726	Cellular response to diacyl bacterial lipopeptide	3	6	2.29

## Data Availability

This is not applicable to this study.

## Supplementary Materials

The following supporting information can be downloaded at: https://www.mdpi.com/article/10.3390/jpm12061007/s1, Figure S1: Each well in an RT2 Profiler PCR Array measures the expression of named 84 genes related to Toll like Receptor pathways; Figure S2: Relative fold change between sham healthy and burn injury using the PARN-018ZD RT2 Profiler PCR Array. Table S1: Analysis of all DEGs using String-DB.com; Table S2: Analysis of up-regulated DEGs using https://String-DB.com; Table S3: Analysis of down-regulated DEGs using https://String-DB.com accessed on 6 June 2021; Table S4: protein-protein interactions of up-regulated DEGs; Table S5: protein-protein interactions of down-regulated DEGs.

## Translational Relevance

These findings demonstrate the utility of microarray in burn injury studies, and elucidate possible molecules, as well as related pathways, for targeting novel interventions in burn-induced cardiac dysfunction.

## Abbreviations

DAMP	damage-associated molecular pattern
DEG	differentially expressed gene
DEP	differentially expressed protein
Hpb	hours post-burn
Hmgb1	high-mobility group box 1
IL	interleukin
I/R	ischemia/reperfusion
MAP4K4	mitogen-activated protein kinase kinase kinase kinase-4
PAMP	pathogen-associated molecular pattern
PPI	protein–protein interaction
PRR	pattern-recognition receptor
Ripk2	receptor-interacting serine-threonine protein kinase 2
TBK1	TANK-binding kinase 1
TBSA	total body surface area
TLR	Toll-like receptors
Ube2n	ubiquitin-conjugating enzyme E2 N
Ube2v1	ubiquitin-conjugating enzyme E2 variant 1
