# Supplementary material for: Regulation of Key Immune-Related Genes in the Heart Following Burn Injury
Source: J Pers Med. 2022 Jun 20;12(6):1007. doi: 10.3390/jpm12061007 (PMC9224557; doi:10.3390/jpm12061007)
Supplement: Supplementary file 1 [file jpm-12-01007-s001.zip › jpm-1707118 Supplementary/Figure S1 and S2_JPM.pptx]

## Slide 1
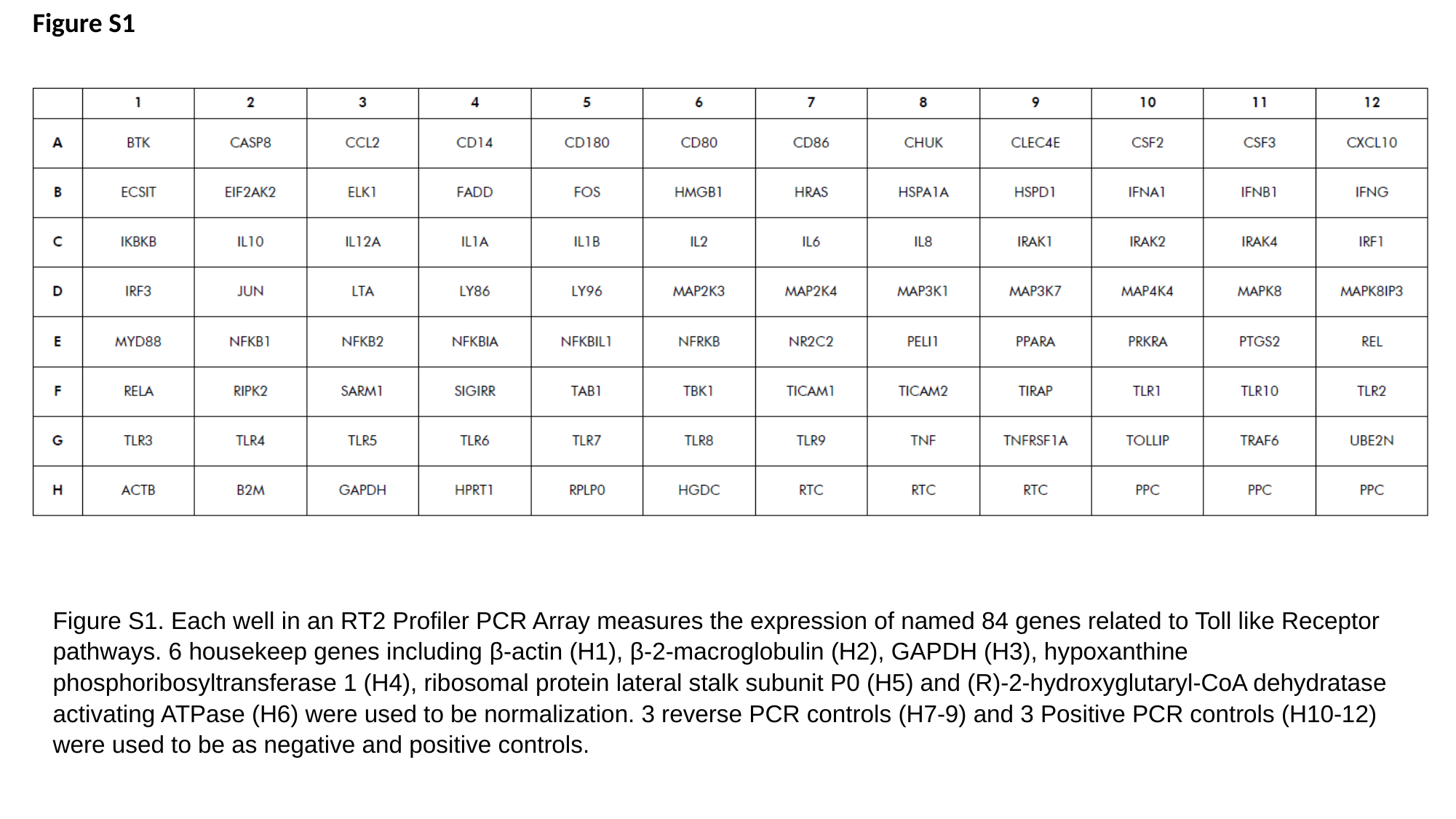

Figure S1
Figure S1. Each well in an RT2 Profiler PCR Array measures the expression of named 84 genes related to Toll like Receptor pathways. 6 housekeep genes including β-actin (H1), β-2-macroglobulin (H2), GAPDH (H3), hypoxanthine phosphoribosyltransferase 1 (H4), ribosomal protein lateral stalk subunit P0 (H5) and (R)-2-hydroxyglutaryl-CoA dehydratase activating ATPase (H6) were used to be normalization. 3 reverse PCR controls (H7-9) and 3 Positive PCR controls (H10-12) were used to be as negative and positive controls.

## Slide 2
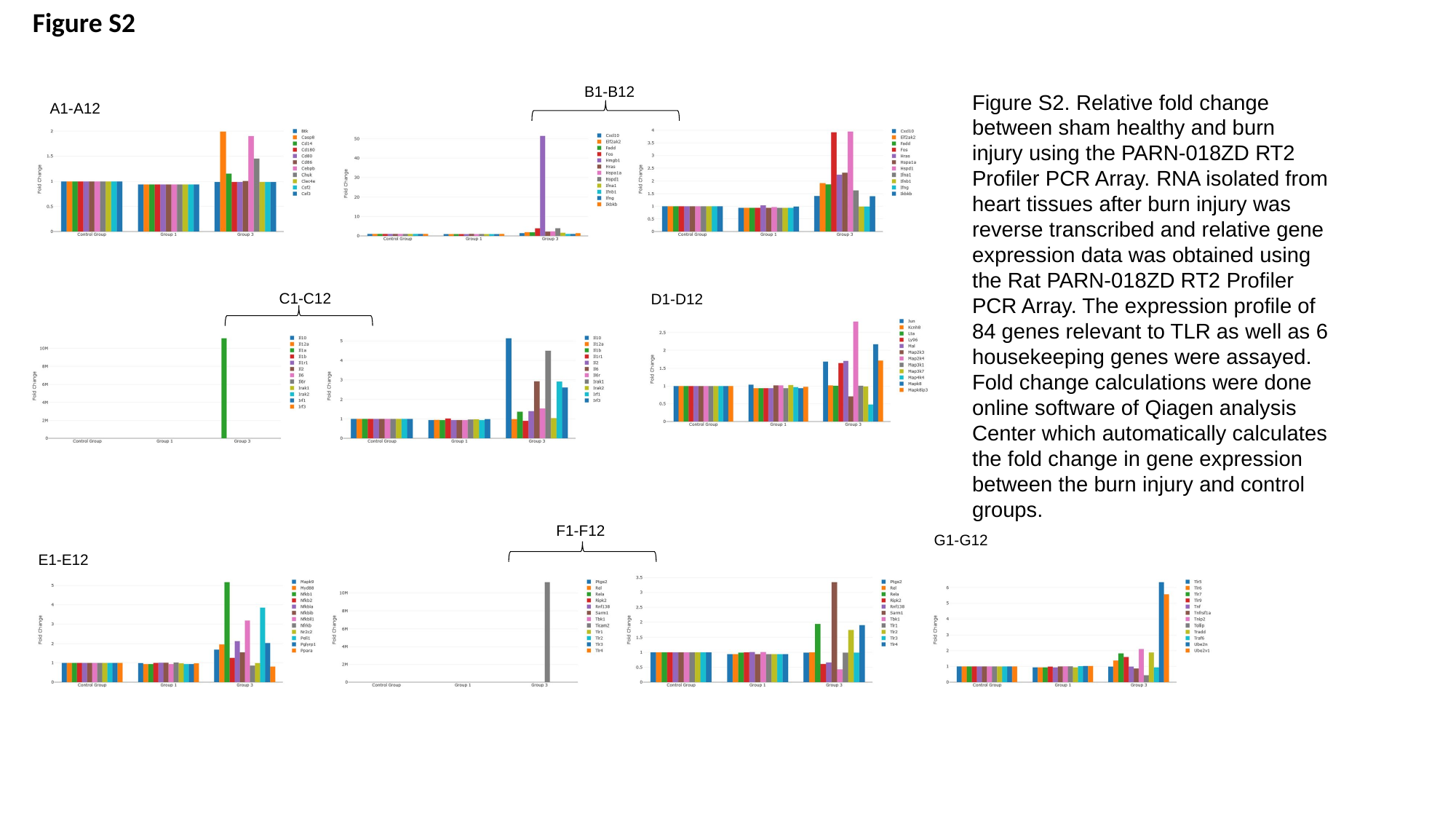

Figure S2
B1-B12
Figure S2. Relative fold change between sham healthy and burn injury using the PARN-018ZD RT2 Profiler PCR Array. RNA isolated from heart tissues after burn injury was reverse transcribed and relative gene expression data was obtained using the Rat PARN-018ZD RT2 Profiler PCR Array. The expression profile of 84 genes relevant to TLR as well as 6 housekeeping genes were assayed. Fold change calculations were done online software of Qiagen analysis Center which automatically calculates the fold change in gene expression between the burn injury and control groups.
A1-A12
C1-C12
D1-D12
F1-F12
G1-G12
E1-E12
